# CRISPR/Cas9-mediated gene knockout and interallelic gene conversion in human induced pluripotent stem cells using non-integrative bacteriophage-chimeric retrovirus-like particles

**DOI:** 10.1186/s12915-021-01214-x

**Published:** 2022-01-07

**Authors:** Joffrey Mianné, Amel Nasri, Chloé Nguyen Van, Chloé Bourguignon, Mathieu Fieldès, Engi Ahmed, Christine Duthoit, Nicolas Martin, Hugues Parrinello, Anaïs Louis, Alexandra Iché, Régis Gayon, Florine Samain, Lucille Lamouroux, Pascale Bouillé, Arnaud Bourdin, Said Assou, John De Vos

**Affiliations:** 1 IRMB, Univ Montpellier, INSERM, CHU Montpellier, Hôpital St Eloi, 80 avenue Augustin Fliche, 34295 Montpellier, France; 2 Flash Therapeutics, Toulouse, France; 3 Univ. Montpellier, CNRS, INSERM, Montpellier, France; 4 MGX-Montpellier GenomiX, Univ. Montpellier, CNRS, INSERM, Montpellier, France; 5 PhyMedExp, Univ Montpellier, INSERM, CHU Montpellier, Montpellier, France; 6 Department of Cell and Tissue Engineering, Univ Montpellier, CHU Montpellier, Montpellier, France

**Keywords:** hiPSC, Transduction, CRISPR, Retrovirus-like particles, Gene conversion, Knock-out

## Abstract

**Background:**

The application of CRISPR/Cas9 technology in human induced pluripotent stem cells (hiPSC) holds tremendous potential for basic research and cell-based gene therapy. However, the fulfillment of these promises relies on the capacity to efficiently deliver exogenous nucleic acids and harness the repair mechanisms induced by the nuclease activity in order to knock-out or repair targeted genes. Moreover, transient delivery should be preferred to avoid persistent nuclease activity and to decrease the risk of off-target events. We recently developed bacteriophage-chimeric retrovirus-like particles that exploit the properties of bacteriophage coat proteins to package exogenous RNA, and the benefits of lentiviral transduction to achieve highly efficient, non-integrative RNA delivery in human cells. Here, we investigated the potential of bacteriophage-chimeric retrovirus-like particles for the non-integrative delivery of RNA molecules in hiPSC for CRISPR/Cas9 applications.

**Results:**

We found that these particles efficiently convey RNA molecules for transient expression in hiPSC, with minimal toxicity and without affecting the cell pluripotency and subsequent differentiation. We then used this system to transiently deliver in a single step the CRISPR-Cas9 components (Cas9 mRNA and sgRNA) to generate gene knockout with high indel rate (up to 85%) at multiple loci. Strikingly, when using an allele-specific sgRNA at a locus harboring compound heterozygous mutations, the targeted allele was not altered by NHEJ/MMEJ, but was repaired at high frequency using the homologous wild type allele, i.e., by interallelic gene conversion.

**Conclusions:**

Our results highlight the potential of bacteriophage-chimeric retrovirus-like particles to efficiently and safely deliver RNA molecules in hiPSC, and describe for the first time genome engineering by gene conversion in hiPSC. Harnessing this DNA repair mechanism could facilitate the therapeutic correction of human genetic disorders in hiPSC.

**Supplementary Information:**

The online version contains supplementary material available at 10.1186/s12915-021-01214-x.

## Background

Human pluripotent and induced stem cells (hPSC and hiPSC, respectively) can self-renew, display unlimited proliferative potential, and can differentiate into any cell type of the three germ layers (ectoderm, mesoderm, endoderm). Therefore, they hold tremendous potential not only for basic research and disease modeling, but also for cell production for clinical applications [1, 2]. This potential has been magnified by the development of gene editing tools, particularly those based on clustered regularly interspaced short palindromic repeats (CRISPR) technologies, to genetically manipulate the hPSC genome [3, 4]. This has allowed the generation of numerous lines with isogenic controls that have become invaluable tools for gene function analysis, genetic disorder modeling, and autologous or allogenic tissue generation for cell-based therapies [1, 2].

However, delivering nucleic acids in hPSC for overexpression or genetic engineering is still a challenging task. This can be explained by several factors, including inefficient delivery systems for hPSC, high cell death rates following transfection using standard transfection tools, difficulties to sub-clone these cells, and low tolerance to DNA double-strand breaks (DSB) [4–6]. Despite recent improvements based on new strategies, such as the transfection of ribonucleoprotein complexes, and the development of new gene transfer tools to enhance delivery in hPSC [7–14], new systems are still required to efficiently deliver nucleic acids in hPSC with minimal toxicity.

Particularly, it would be important to develop a system to efficiently deliver RNA molecules for transient overexpression without transgene insertion in the genome. Currently, mRNA molecules can be delivered in hPSC by electroporation and lipofection or via non-viral particles. However, these systems are usually highly toxic and result in low transfection efficacy. Recently, several groups have described non-integrative RNA delivery systems that are based on modified viral particles and that combine the viral vector high transduction efficacy and low toxicity, without genome integration of the viral payload [15–20]. Several of these systems rely on bacteriophage-chimeric retrovirus-like particles to encapsulate concomitantly (but with variable efficiency) different types of RNA molecules, for instance CRISPR-associated protein 9 (Cas9) mRNA and the CRISPR-associated protein 9 (Cas9) necessary for CRISPR/Cas9 gene editing [21, 22]. Therefore, we asked whether one of these systems could be used to deliver RNA molecules for transient overexpression and genome engineering in hiPSC. To this aim, we targeted three genes [multiciliate differentiation and DNA synthesis associated cell cycle protein (*MCIDAS*), and coiled-coil domain containing 40 (*CCDC40*)] implicated in the pathogenesis of primary ciliary dyskinesia (PCD), a rare pulmonary genetic disorder, and two genes with therapeutic interest for T-cell engineering [T cell receptor alpha constant (*TRAC*) and C-X-C chemokine receptor type 4 (*CXCR4*)].

First, we found that bacteriophage-chimeric retrovirus-like particles can very efficiently transduce hiPSC lines with minimal toxicity and no impact on pluripotency. Then, we used this system to simultaneously and efficiently transfer sgRNA and Cas9 mRNA for non-homologous end-joining (NHEJ)/micro-homology end-joining (MMEJ)-based gene editing at multiple loci. Finally, we demonstrated for the first time that interallelic gene conversion is a DNA repair mechanism that can be activated in hiPSC following introduction of a DNA DSB. This mechanism was harnessed to correct a heterozygous mutation in a patient hiPSC line and for converting heterozygous mutations into homozygous mutations.

## Results

### Bacteriophage-chimeric retrovirus-like (LentiFlash®) particles for highly efficient and transient RNA delivery in hiPSC

**Fig. 1:**
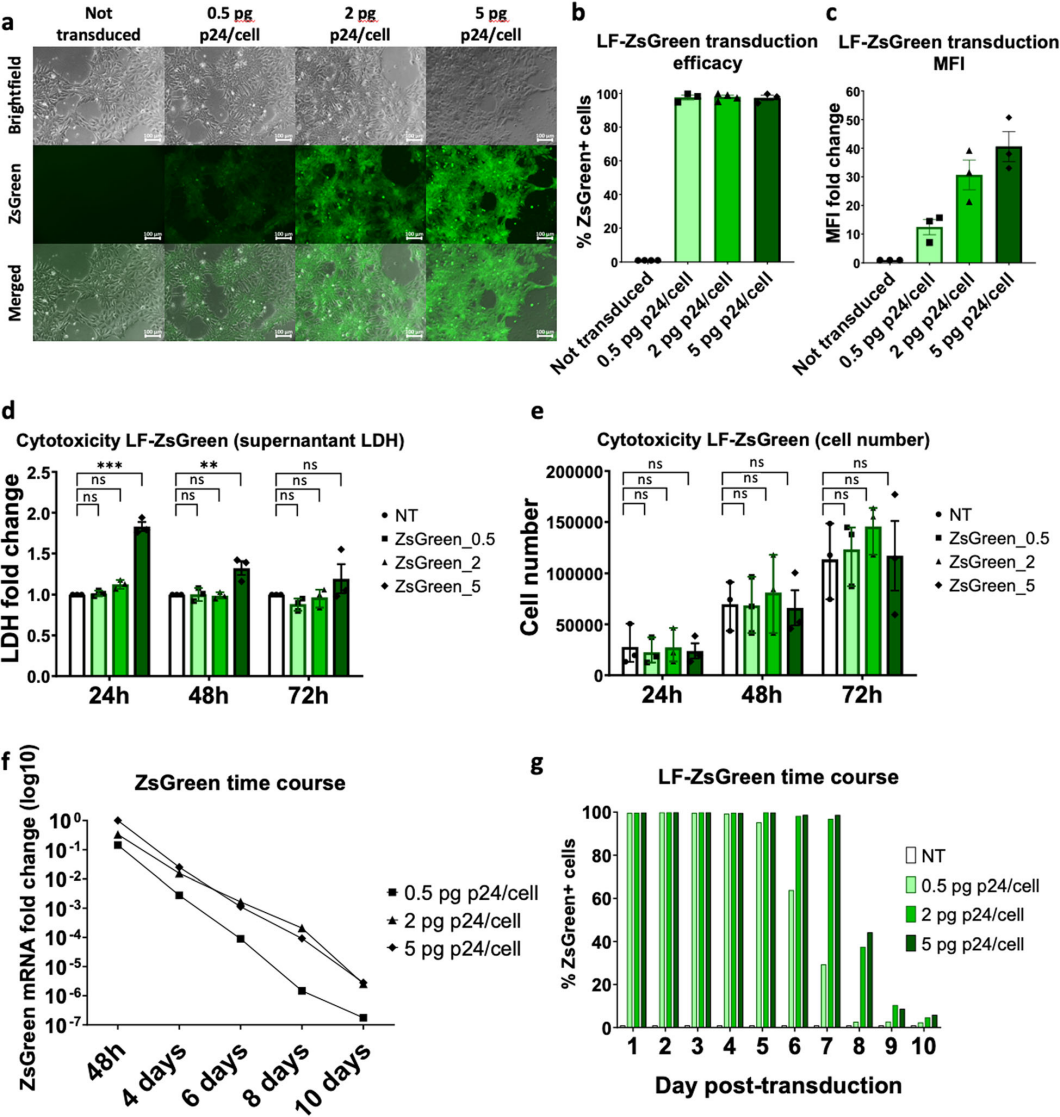
LentiFlash® particles allow the highly efficient delivery of RNA molecules in hiPSC. **a** Fluorescent microscopy analysis of HY03 hiPSC 48 h post-transduction with LF-ZsGreen particles at 0.5, 2, or 5 pg p24/cell. **b** Transduction efficacy by flow cytometry analysis of ZsGreen-positive HY03 cells at 48 h post-transduction, and **c** mean fluorescent intensity (MFI) fold change assessed by cytometry analysis at 48 h post-transduction (same conditions as in **a**). **d** Fold change of lactate dehydrogenase (LDH) concentration in HY03 cell supernatant samples collected at 24, 48, and 72 h post-transduction (same conditions as in **a**); NT, not transduced cells (*n* = 1); ns, not significant; **P < 0.005; ***P < 0.0005. **e** Cell number at 24, 48, and 72 h post-transduction (same conditions as in **a**); NT, not transduced cells (*n* = 1); ns, not significant. **f** Fold change of ZsGreen mRNA level in HY03 cells in function of time. The sample corresponding to cells transduced with 5 pg p24/cell and analyzed at 48 h was arbitrarily defined as the reference (=1) (same conditions as in **a**, *n* = 1). **g** Flow cytometry analysis of ZsGreen-positive cells for 10 days after transduction (NT, not transduced cells)

To investigate whether LentiFlash® particles can transduce hiPSC, we generated particles containing the fluorescent reporter ZsGreen mRNA (LF-ZsGreen) and used them at three doses (0.5, 2, and 5 pg p24/cell) to transduce a hiPSC line (HY03) derived from a healthy individual [23]. Analysis of ZsGreen expression by fluorescence microscopy and flow cytometry at 48 h post-transduction revealed that more than 97% of cells were transduced (all three tested doses) and that fluorescence signal intensity was dose-dependent (*n* = 3) (Fig. 1a–c). Moreover, cell counting and lactate dehydrogenase (LDH; a marker of cytotoxicity) quantification in supernatant confirmed the low toxicity observed by optical microscopy, without significant increase in LDH concentration or cell loss after transduction with particles at the concentration of 0.5 and 2 pg p24/cell. We observed only a slight increase in LDH concentration at the highest dose tested (5 pg p24/cell), without any effect on cell proliferation (Fig. 1d, e). ZsGreen expression monitoring by real-time quantitative PCR (qPCR) and flow cytometry analysis for 10 days revealed the transient nature of the system with a sharp decline in ZsGreen mRNA level over time, followed by a progressive decrease in ZsGreen protein expression (Fig. 1f, g). This decrease was dose-dependent and reached control levels at day 10. The percentage of ZsGreen-positive cells peaked at 24 h post-transduction (all three doses), whereas the fluorescence signal intensity reached its maximum at 48 h (Fig. 1 g, and Fig. S1a-b). These findings confirmed previous data obtained in HeLa cells (data not shown) and clearly show that we can specifically quantify ZsGreen RNA (by qPCR) and ZsGreen protein expression (by cytometry) in cells transduced with LentiFlash® particles. The detection of ZsGreen RNA demonstrates that the RNA contained in the LentiFlash® particles was efficiently transferred in the transduced cells. These experiments also showed that while ZsGreen RNA expression decreased already at 48 h after transduction, ZsGreen protein levels remained high for 5–10 days (in function of the used dose) because the protein half-life is quite long. These results suggest that after transduction of LentiFlash® particles, protein expression in target cells is correlated with the RNA delivery. Transduced hiPSC retained a pluripotent morphology and expressed pluripotency markers, as indicated by the expression of NANOG, OCT3/4, SOX2, and SSEA4 (Fig. S1c). Pluripotency was confirmed by differentiating transduced cells into definitive endoderm that expressed CXCR4, FOXA2, and SOX17 (Fig. S1d). We replicated these results in two additional hiPSC lines, one derived from a patient with PCD (PCD_02:30 line) [24] and the other from a patient with chronic obstructive pulmonary disease (COPD) (iCOPD9_B27 line) (Fig. S2).

### Efficient CRISPR/Cas9-mediated NHEJ/MMEJ genome editing in hiPSC

**Fig. 2:**
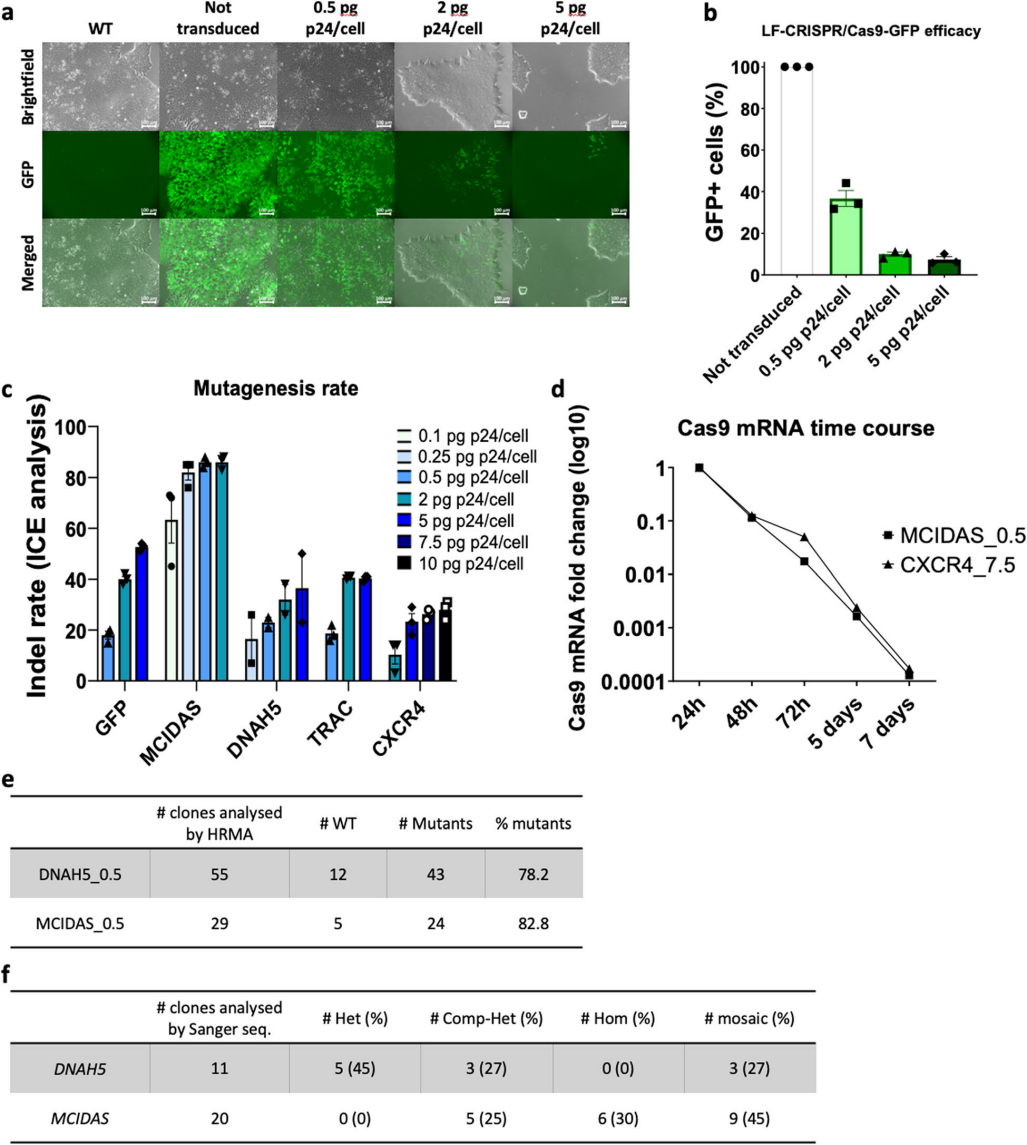
The LentiFlash® system can be used for the efficient CRISPR/Cas9-mediated modification of hiPSC. **a** Fluorescent microscopy analysis of HY03-GFP cells at day 6 post-transduction with LF-CRISPR/Cas9-GFP particles at 0.5, 2, or 5 pg p24/cell. Wild-type cells (WT) are parental HY03 cells. **b** Transduction efficacy measured by flow cytometry quantification of GFP-positive HY03-GFP cells at day 6 post-transduction with LF-CRISPR/Cas9-GFP particles at 0.5, 2, or 5 pg p24/cell (NT, not transduced = 100%). **c** Indel rate at five loci (*GFP*, *MCIDAS*, *DNAH5*, *TRAC*, and *CXCR4*) using increasing doses of LentiFlash® particles harboring CRISPR/Cas9 to target the indicated genes (from 0.1 to 10 pg p24/cell) in HY03 hiPSC cells at day 3 post-transduction. Data were obtained by ICE decomposition analysis after Sanger sequencing of the targeted loci. **d** Fold change of Cas9 mRNA level in HY03 cells transduced with LF-CRISPR/Cas9-MCIDAS or LF-CRISPR/Cas9-CXCR4 (0.5 and 7.5 pg p24/cell, respectively). The samples corresponding to cells analyzed at 24 h were arbitrarily defined as the reference (=1) (*n* = 1) **e** HRMA results of clones obtained following HY03 cells transduction with LF-CRISPR/Cas9-DNAH5 and LF-CRISPR/Cas9-MCIDAS particles at 0.5 pg p24/cell. WT, wild type. **f** Sanger sequencing analysis of clones identified as mutated by HRMA. Het, heterozygote; Comp-Het, compound heterozygote; Hom, homozygote

Then, we asked whether LentiFlash® particles could be used to transiently deliver CRISPR/Cas9 reagents in hiPSC for genome engineering. To this aim, we first generated a non-clonal GFP-reporter stable cell line (HY03-GFP) by transducing HY03 cells with integrative recombinant lentiviral particles (ILV-EF1-GFP) (Fig. S3). Then, we transduced HY03-GFP cells with LentiFlash® particles that carry a sgRNA targeting the *GFP* sequence and the Cas9 mRNA (LF-CRISPR/Cas9-GFP) at three doses (0.5, 2, and 5 pg p24/cell) to assess NHEJ/MMEJ-mediated *GFP* knock-out. Six days post-transduction, fluorescent microscopy and flow cytometry analysis showed that GFP expression was absent in more than 90% of cells at the highest dose tested (Fig. 2a, b). Sanger sequencing and Interference of CRISPR Edits (ICE) analysis confirmed the high frequency of NHEJ/MMEJ mutations at the targeted *GFP* sequence (Fig. 2c).

Then, we generated four additional LentiFlash® vectors (LF-CRISPR/Cas9-MCIDAS, LF-CRISPR/Cas9-DNAH5, LF-CRISPR/Cas9-TRAC, and LF-CRISPR/Cas9-CXCR4) to target two endogenous genes involved in motile ciliary biology (*MCIDAS* and *DNAH5*) and two endogenous genes implicated in T-cell biology (*TRAC* and *CXCR4*). Transduction of HY03 hiPSC with increasing doses resulted in dose-dependent NHEJ/MMEJ-mediated mutagenesis at all four loci, as indicated by Sanger sequencing and ICE analysis. The indel rates obtained were gene-dependent, with mutagenesis rates of 86, 36, 40, and 28% for *MCIDAS*, *DNAH5*, *TRAC*, and *CXCR4*, respectively (Fig. 2c). For DNAH5, in line with the ICE analysis, NGS-based amplicon sequencing showed that 49.5% of reads displayed deletions, confirming a high efficiency of CRISPR/Cas9 editing (Fig. S4). As seen for ZsGreen expression, Cas9 mRNA level monitoring by real-time qPCR for 7 days confirmed the rapid mRNA decline after LF-CRISPR/Cas9-MCIDAS (0.5 pg p24/cell) or LF-CRISPR/Cas9-CXCR4 (7.5 pg p24/cell) transduction (Fig. 2d). This is particularly interesting for limiting Cas9 activity over time and reducing potential off-target mutations. To demonstrate that Cas9 mRNA and sgRNA are co-packaged in LentiFlash® particles, we assessed CRISPR/Cas9 targeting efficiency using various batches of LentiFlash® particles to edit the GFP gene in a clonal HCT116 cell line that contains one copy of GFP per cell. Specifically, we analyzed GFP expression at day 7 after transduction of LentiFlash® particles that express (i) Cas9-MS2 alone, (ii) sgRNAGFP-PP7 and Cas9 without the MS2 aptamers (two different plasmids), (iii) sgRNAGFP-PP7 and Cas9-MS2 (two different plasmids), and (iv) sgRNAGFP-PP7 and Cas9-MS2 (both in the same expression plasmid, which corresponds to the LentiFlash® generation process used for the iPSC experiments described in this article). We observed 75% of GFP editing when cells were transduced with sgRNAGFP-PP7 or Cas9 without the MS2 aptamers. This indicated that some Cas9 proteins are passively packaged or embedded with the guide packaging. When Cas9 RNA is actively packaged, the editing efficiency increased to more than 90% (supplementary Fig. S5). As expected, we obtained the best editing efficiency (97.7%) using LentiFlash® particles produced with a single expression plasmid that allows the active packaging of sgRNA-PP7 and Cas9-MS2. These data show that although the Cas9 protein is passively packaged during the production process, the CRISPR/Cas9 system is more efficient when Cas9 is actively packaged thanks to the presence of MS2 aptamers.

Sub-cloning of cells transduced with 0.5 pg p24/cell LF-CRISPR/Cas9-DNAH5 or LF-CRISPR/Cas9-MCIDAS particles and screening by high-resolution melting analysis (HRMA) confirmed gene editing, with 78% and 82% of clones mutated at the *DNAH5* and *MCIDAS* locus, respectively (Fig. 2e). Sanger sequencing of *DNAH5* and *MCIDAS* in 11 and 20 randomly selected mutant clones (according to the HRMA results) confirmed the targeted mutagenesis (Fig. 2f). Furthermore, the characterization of two knock-out cell lines (one for *DNAH5* and one for *MCIDAS*) confirmed the expression of pluripotency markers (NANOG, OCT3/4, SOX2, and SSEA4), the absence of the most recurrent genomic abnormalities found in hiPSC, and the absence of Cas9 genomic integration (Fig. S6).

Finally, to confirm the data reproducibility, we performed CRISPR/Cas9 editing in the other two hiPSC lines (PCD_02:30 and iCODP9_B27) using optimized particle concentrations. This resulted in comparable mutagenesis rates in all cell lines for the four targeted genes (Fig. S7).

### Allele-specific gene editing induces interallelic gene conversion in hiPSC

**Fig. 3:**
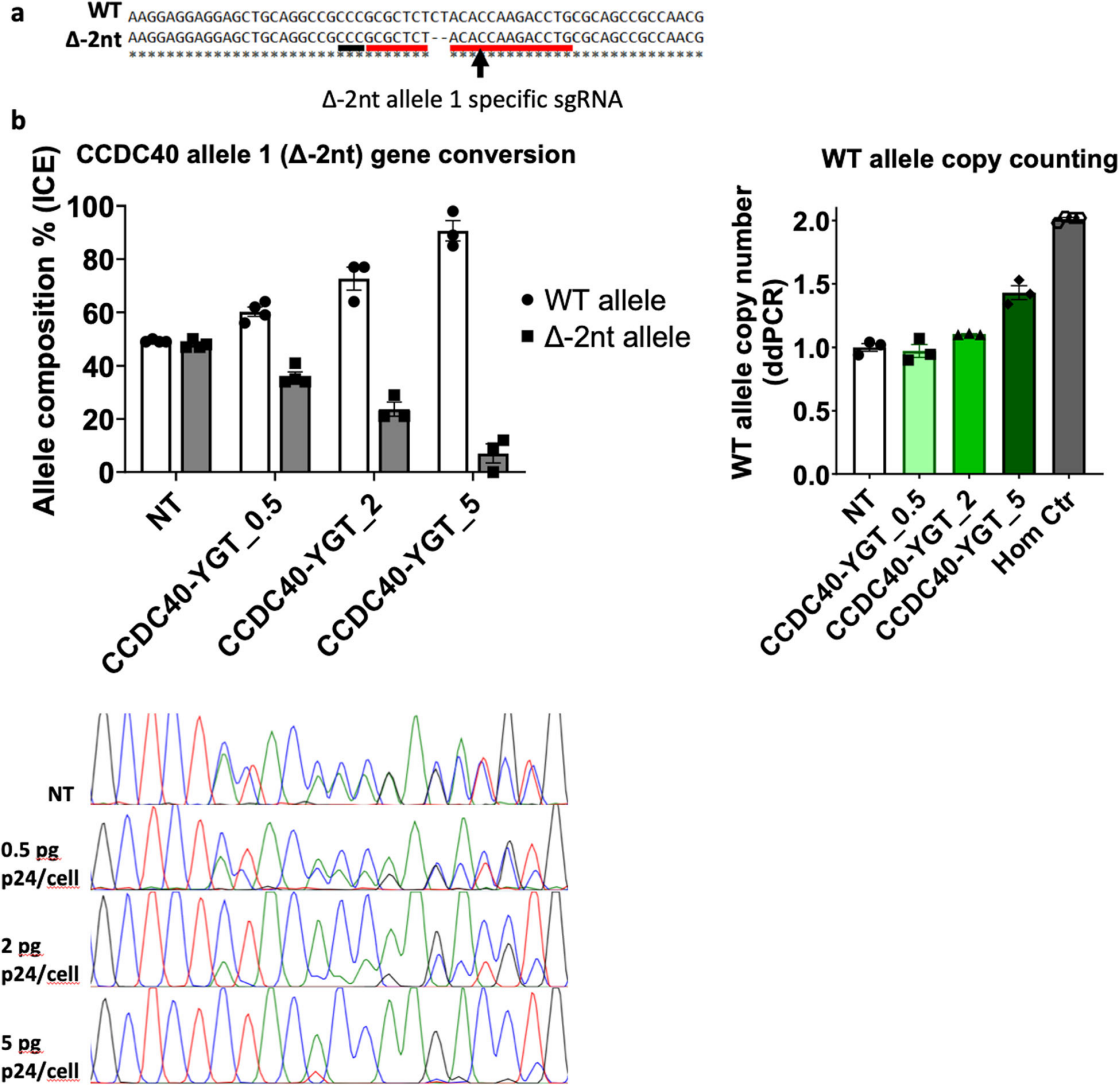
Allele-specific targeting of a pathogenic allele results in gene conversion. **a** Allele-specific sgRNA design strategy to target the *CCDC40* allele 1 (Δ-2nt) in the PCD_02:30 hiPSC line. The protospacer sequence is underlined in red, and the protospacer adjacent motif in black. **b** Targeting of Δ-2nt allele by transducing PCD_02:30 cells with LF-CRISPR/Cas9-CCDC40-YGT particles at 0.5, 2, and 5 pg p24/cell results in dose-dependent interallelic gene conversion. Top left: Allelic composition at the targeted locus. Top right: Wild type (WT) allele copy number was measured by droplet digital PCR (ddPCR). Bottom: Representative Sanger sequencing chromatograms. ICE, Inference of CRISPR Edits; Hom, homozygote; NT, not transduced

The PCD_02:30 hiPSC line was derived from a patient with PCD harboring *CCDC40* compound heterozygous mutations [24]. The two heterozygous pathogenic variants correspond to a 2-nucleotide frameshift deletion in exon 7 (Δ-2nt, allele 1) and a single nucleotide polymorphism in the splice site acceptor of exon 19 (G > A, allele 2). To investigate whether a CRISPR/Cas9 reframing approach could restore the reading frame in allele 1, we designed an allele-specific sgRNA that encompasses in its seed region the Δ-2nt variant (Fig. 3a). We then used this sgRNA to generate the corresponding LentiFlash® particles (LF-CRISPR/Cas9-CCDC40-YGT). Transduction of the PCD_02:30 hiPSC line did not lead to the generation of indels at the targeted locus, but decreased the percentage of Δ-2nt alleles and increased the percentage of wild type allele (WT) at this position, as indicated by Sanger sequencing and ICE analysis (Fig. 3b). These results were reproducible (*n* = 3) and dose-dependent, with an increase of the WT allele percentage from 50% (heterozygous state) to 90% after transduction with LF-CRISPR/Cas9-CCDC40-YGT particles at 5 pg p24/cell. To determine whether these results were due to larger deletions of the targeted Δ-2nt allele with a resulting over-representation of the WT sequence, we counted the copies of the WT allele by droplet digital PCR (ddPCR). In agreement with the Sanger sequencing data, the WT allele copy number was increased in a dose-dependent manner (*n* = 3), with a mean copy number of 1.43 (equivalent to 71% of WT alleles compared with 90% by ICE analysis) for the 5 pg p24/cell condition (Fig. 3b). This confirmed that the targeted Δ-2nt allele was corrected to a WT allele. To confirm that our sgRNA design was allele-specific, we transduced the HY03 hiPSC line (WT at the *CCDC40* locus) with LF-CRISPR/Cas9-CCDC40-YGT particles at 5 pg p24/cell. We did not detect any indel by Sanger sequencing, confirming the sgRNA specificity (data not shown). This finding was not specific to the use of the LentiFlash® system because we obtained interallelic gene conversion also by electroporating the PCD_02:30 iPSC line with CRISPR/Cas9 RNPs targeting the Δ-2nt allele, albeit at a lower efficiency (interallelic gene conversion occurring, on average, in 42% of cells) (Fig. S8a). Of note, the electroporation conditions must be optimized for each cell line. Indeed, the conditions used to electroporate PCD_02:30 iPSC resulted in complete loss of HY03 hiPSC viability (data not shown). Therefore, we concluded that allele-specific targeting of the Δ-2nt pathogenic variant resulted in DNA repair mediated by interallelic gene conversion.

To determine whether this phenomenon was conserved upon loss of pluripotency, we differentiated PCD_02:30 hiPSC into neural progenitors (Fig. S8b). Transduction of these progenitors with LF-CRISPR/Cas9-CCDC40-YGT at 0.5, 2, and 5 pg p24/cell gave similar results as those obtained with the parental hiPSC line: increase of the WT allele percentage from 50 (heterozygous state) to 90% after transduction at the highest dose. This suggests that interallelic gene conversion occurs also after hPSC differentiation and loss of pluripotency (Fig. S8c).

Finally, to determine whether interallelic gene conversion could occur at another locus and more generally whether this genome engineering approaches could be harnessed to convert heterozygous mutations to homozygous mutations, we derived three heterozygous clonal lines with a frameshift mutation in *DNAH5* (DNAH5_A6, DNAH5_C3, and DNAH5_B5; Fig. S9). These clones were obtained by transducing the HY03 hiPSC line with LF-CRISPR/Cas9-DNAH5 (see above), and harbored one allele with an indel (7nt deletion [Δ-7nt], 1nt insertion [Δ+1nt], or a combination of 1nt deletion with 2nt insertion [Δ-1/+2nt] for DNAH5_A6, DNAH5_C3, and DNAH5_B5, respectively) that disrupted the sgRNA binding site, and a WT allele that could be targeted to introduce a DSB. Transduction with LF-CRISPR/Cas9-DNAH5 at the optimized dose of 0.5 pg p24/cell showed the absence of off-target mutations at sites containing up to three mismatches and the absence of the most recurrent genomic abnormalities found in hiPSC (Fig. S9). It also resulted in gene conversion with an increase of the mutant allele copy number specific to each clone (the Δ-7nt for DNAH5_A6, Δ+1nt for DNAH5_C3 and Δ-1/+2nt for DNAH5_B5), as indicated by Sanger sequencing and ICE analysis (Fig. S10). These results confirmed that allele-specific targeting in hiPSC results in DNA repair mediated by interallelic gene conversion.

## Discussion

Our data show that LentiFlash® particles very efficiently deliver non-integrative RNA molecules in hiPSC. We demonstrated that this system can be used to transiently express RNA molecules in up to 97% of transduced cells. Moreover, this highly efficient transduction system does not cause marked cytotoxicity and thus allows reducing the number of cells to be used and performing experiments directly after transduction, without the need of an extended recovery period. This could be particularly interesting for many applications, including the expression of selected transcription factors to direct hPSC differentiation.

Furthermore, thanks to its versatility, this system can be used to deliver simultaneously various RNA types, including the Cas9 mRNA and sgRNA for CRISPR/Cas9-based gene editing. Our result shows that the LentiFlash® technology allows packaging both the Cas9 RNA and the Cas9 protein, especially because the corresponding encoding DNA is efficiently transfected in the producer cells. The editing rates we obtained in HCT116 cells that contain one copy of the target gene per cell cannot be extrapolated to the level of expression of endogenous genes where many factors can interfere with the editing efficiency (gene copy number, availability of the gene within chromatin, sgRNA design). In fact, a small amount of Cas9 protein might efficiently edit a gene present as a single copy per cell, but be ineffective in other gene configurations [25]. We demonstrated that LentiFlash® particles can be exploited to efficiently manipulate the hiPSC genome via the NHEJ/MMEJ repair pathways. By targeting five genes in three different hiPSC lines, we could generate indels in up to 85% of the targeted alleles. Moreover, we did not observe off-target events at any of the loci analyzed in the five stable cell lines generated in this study.

Thanks to the use of the active cellular entrance pathway of classical lentiviral vectors, LentiFlash® delivery does not disrupt cell integrity, differently from other transfection systems, such as electroporation and lipofection. This results in very low cell toxicity and minimal cell death after transduction (Fig. 1d, e). This easy-to-use technology is also very efficient (> 97% of cells expressed the transgene of interest) (Fig. 1), thus saving time and work linked to cell selection or sorting. Recent studies showed that lentivirus-like particles (LVLP) can be generated with an approach similar to the one described in the present work to obtain a CRISPR/Cas9 system using saCas9, SpCas9, or adenine-based editing systems [15, 26, 27]. The design of these LVLP (number and location of the MS2 coat protein, number of MS2 aptamer repeats, and 3′UTR modification) was different from ours. Like these authors, we observed that LentiFlash® particles were morphologically similar to conventional lentiviral vectors [20]. Differently from us, these authors reported that the viral titers were decreased when MCP was cloned into the matrix gene. Indeed, the titers we obtained with these vector batches remained high, and they are in the best design configuration for efficient gene editing. Other groups used similar bacteriophage systems to create particles that can transfer RNA into target cells using a slightly different design compared with ours [15, 18, 19]. Briefly, Knopp et al. replaced the nucleocapsid by a genetically fused MS2 heterodimer to generate non-integrating gammaretroviral murine leukemia virus-based CRISPR/Cas9 all-in-one particles. Lindel et al. used a transient prototype foamy virus (PFV) vector system to deliver Cas9 mRNA combined with an integration-deficient retroviral vector (IDRV) to carry the sgRNA, allowing efficient gene editing. They also showed that sgRNA delivery using the transient PFV vector was less efficient than with the IDRV vector, but resulted in significant editing rates when transduced 24 h after the Cas9 mRNA-carrying transient PFV. Although LentiFlash® particles contain RNPs, sgRNA and RNA for Cas9 are actively encapsidated via the MS2 coat protein-aptamer interaction. We think that some editing activity can be attributed to passively packaged or embedded Cas9 protein and that this activity might be maintained by the expression of Cas9 RNA. This might explain the increased editing efficiency when using the active packaging system. LentiFlash® particles allow the delivery of RNA molecules in the cell cytoplasm, without any viral sequence, thus completely avoiding reverse transcription and integration in the cell genome (Fig. S6e). Therefore, LentiFlash® particles could be an interesting tool for transient expression for gene overexpression, gene editing, or transcription factor-based differentiation.

Considering the high capacity of LentiFlash® particles to efficiently deliver CRISPR/Cas9 reagents at multiple loci, we investigated whether this system could be used in a reframing strategy at a pathogenic locus. Therefore, we designed a sgRNA specific for a pathogenic variant in a patient-derived hiPSC line, to introduce indels in the close vicinity of this frameshift mutation. To our surprise, this did not result in the generation of indels, but in interallelic gene conversion that allowed correcting the variant to a WT sequence in more than 40% of transduced cells. Interestingly, this phenomenon was conserved following hiPSC differentiation to neural progenitors, suggesting that it is not restricted to pluripotent cells. Furthermore, we confirmed gene conversion after allele-specific targeting of a frameshift mutation at an additional locus (*DNAH5*) in three clones derived from a second hiPSC line (HY03). To the best of our knowledge, interallelic gene conversion has never been described in hPSC.

Gene conversion is a form of homologous recombination that is initiated by DNA DSB and results in the unidirectional transfer of genetic material from the intact homologous sequence to the region that contains the DSB. This phenomenon is well documented during meiosis and is a recognized driver not only of human genome evolution, but also of inherited diseases. However, it is also involved in the occasional phenotype reversion of genetic diseases, such as epidermolysis bullosa [28]. More recently, this DNA repair mechanism was described in other cell types after CRISPR/Cas9-mediated DNA DSB. One report demonstrated interallelic gene conversion in cultured human cells, including human embryonic kidney cells (HEK293T) and human primary lung stromal cells [29], but only in 2.3% and 0.15% of the alleles, respectively. Two other reports described interallelic gene conversion in mouse embryonic stem cells, with different yields: 4 × 10^− 5^ in the study by Susani et al. [30] and 40% in the study by Wu et al. [31]. As we did in our study, Wu et al. used an allele-specific sgRNA that included the variant sequence in the protospacer adjacent motif. Finally, several articles described this phenomenon after CRISPR/Cas9 injection in early embryos. Although interallelic gene conversion in embryos has been controversial [32–35], this DNA repair mechanism has been shown to be activated in mouse [31], rat [36], and human embryos [37–39]. Although the embryo number and targeted loci remain low, interallelic gene conversion following CRISPR/Cas9 mutagenesis seems to occur more frequently in embryos: 28% in the rat (2/7 embryos) [36], 18% in the mouse (4/22 embryos) [31], and up to 40% in human embryos [37, 38]. Moreover, this DNA repair pathway can be modulated through the use of exogenous molecules. For instance, the 53BP1 inhibitor i53 increases gene conversion in different human and mouse cell lines [40]. Moreover, RAD51 was recently used to increase interallelic gene conversion in mouse embryos from 26 to 74% by Wilde et al. [41]. These studies indicate that interallelic gene conversion might be a conserved DNA repair mechanism activated at high frequency during early mammalian embryogenesis and consequently also in pluripotent stem cells. As DNA damage response can be cell-type specific [42], and the DNA repair response is more efficient in pluripotent stem cells than in somatic cells [43, 44], we investigated whether interallelic gene conversion is also active beyond the pluripotency stage. We obtained similar results in neural progenitors derived from the same hPSC line, suggesting that this phenomenon is at least conserved in some cell types.

## Conclusions

Our data show, for the first time, the occurrence of interallelic gene conversion in hiPSC after CRISPR/Cas9 induced DSB. This repair process is observed at a high rate only when one allele is specifically targeted. Interallelic gene conversion in hiPSC could be used to efficiently engineer their genome without donor DNA template. Moreover, unraveling the molecular machinery responsible for gene conversion in hiPSC could lead to the development of strategies to modulate and increase this DNA repair pathway in differentiated cells. This could provide a simple way to convert heterozygote alleles to a homozygous state at targeted loci, opening the way to correct human recessive genetic disorders caused by compound heterozygous mutations and human autosomal dominant genetic disorders.

## Methods

### hiPSC lines, culture, and passaging

Three hiPSC lines reprogrammed in the laboratory were used: (i) HY03 (alternative name listed in the hPSCReg registry: UHOMi002-A), derived from an healthy individual [23]; (ii) PCD_02:30 (alternative name listed in the hPSCReg registry: UHOMi001-A), derived from a patient with primary ciliary dyskinesia harboring compound heterozygous mutations in the *CCDC40* gene [24]; and (iii) iCOPD9_B27, derived from a patient with chronic obstructive pulmonary disease (COPD) [45]. The three hiPSC lines were maintained in undifferentiated state in feeder-free conditions on growth factor-reduced Geltrex matrix (Thermo Fisher Scientific) in Essential 8™ (E8) medium (Thermo Fisher Scientific) at 37 °C in a humidified atmosphere of 5% CO_2_ in air. Cells were dissociated using Versene (Thermo Fisher Scientific) for single-cell passaging every 4–5 days (when cells reached 70–80% confluence) followed by plating (1:10 to 1:20 ratio) with 10 μM of the ROCK inhibitor Y-27632 (Tocris). The E8 medium was changed daily.

### sgRNA design and selection

*DNAH5* and *MCIDAS* sgRNAs were selected to target a sequence in an exon shared by all the gene transcripts and to be as specific as possible using the freely available CRISPOR online tool (http://crispor.tefor.net/) [46]. For the *CCDC40* reframing/gene conversion experiment, the sgRNA was selected using CRISPOR online tool in order to include the “Δ-2nt, allele 1” mutation [c.1116_1117delCT, p.Y378Hfs] and to produce a DNA DSB as close as possible to the mutation. The sgRNAs targeting *TRAC*, *CXCR4*, and *GFP* were previously described [47–49]. The sgRNA sequences are listed in Supplementary Table S1.

### Integrative lentiviral vector particle production, purification, and quantification by quantitative PCR

Three plasmids were used to produce the recombinant lentiviral particles ILV-EF1-GFP. A first plasmid pLV-GagPol (wt) provided a nucleic acid encoding viral gag and pol genes lacking vif, vpr, vpu, and nef genes. A second plasmid pVSVG provided a nucleic acid encoding the vesicular stomatitis virus envelope glycoprotein (VSV-G). A third self-inactivating expression plasmid encoded the GFP fluorescent reporter under the control of the human elongation factor 1 alpha promoter (EF1a) (Fig. S11). Viral particles were produced in a 10-layer CellSTACK chamber (6360cm^2^, Corning) after transfection of the three plasmids in HEK293T cells using the standard calcium phosphate procedure. Twenty-four hours post-transfection, the supernatant was discarded and replaced by fresh medium and cells were incubated at 37 °C in a humidified atmosphere of 5% CO_2_ in air. After medium change, supernatant was collected, clarified by centrifugation at 3000 *g* for 5 min, and microfiltered through 0.45-μm pore size sterile filter units (Stericup, Millipore). Supernatant was harvested several times, and finally, all samples were pooled (crude harvest). The crude harvest was concentrated and purified by ultrafiltration and diafiltration.

Transduction unit (TU) titration assays of the pooled supernatants were performed as follows. HCT116 cells were seeded in 96-well plates. Twenty-four hours later, transduction was performed using pooled supernatants and the rLV-EF1-GFP internal standard (six serial dilutions). Three days post-transduction, cells were trypsinized and the titer (TU/ml) was determined by qPCR after extraction of genomic DNA using the Nucleospin tissue gDNA extraction kit (Macherey-Nagel). The titer determined by qPCR and expressed in TU/ml was normalized to the internal standard, the titer of which was previously determined by FACS.

### MS2 and PP7-chimeric retrovirus-like particle (LentiFlash®) production, purification, and quantitation by p24 ELISA assay

The non-integrative LentiFlash® particles (Flash Therapeutics) that exploit the bacteriophage MS2 or PP7-Coat and its cognate RNA aptamer were previously described by Prel et al. [20]. Like for the integrative lentiviral particles, three plasmids were used to produce recombinant LentiFlash® particles in HEK293T cells: (i) the pLV-GagPol plasmid encoding the viral gag and pol genes modified to harbor the MS2 and/or PP7-Coat within the gag gene and referred to as pLF-GagPol ZF_PCP and pLF-GagPol MA_MCP.ZF_PCP. pLF-GagPol ZF_PCP was generated using the p8.74 ∆ZF_MS2Coat construct, described by Prel et al. [20], by replacing the MS2 coat protein sequence with the capsid assembly-deficient PP7 coat protein PP7ΔFG sequence described by Chao et al. [50]. The human codon-optimized PP7ΔFG coding sequence was synthesized by Geneart (Thermo Fisher Scientific) and cloned, using the In-Fusion® cloning kit (Takara Bio), into p8.74 ∆ZF_MS2Coat previously digested with *Hpa*I. pLF-GagPol MA_MCP.ZF_PCP was generated by inserting the MS2 coat protein coding sequence into the pLF-GagPol ZF_PCP plasmid between the codons corresponding to the 127th and 128th amino acid of the MA protein using the In-Fusion® cloning kit (Takara Bio) after PCR amplification of the two fragments; (ii) the pVSVG plasmid encoding the VSV-G glycoprotein; and (iii) the plasmid encoding the RNA cargo, flanked by the bacteriophage aptamers to enable RNA mobilization into lentiviral particles through the interaction with the corresponding coat protein cloned in the Gag sequence. For the ZsGreen reporter, the ZsGreen mRNA was flanked by PP7 aptamers on the RNA transfer plasmid as shown in supplementary Fig. S11, and the pLF-GagPol ZF_PCP was used for transfection of the produced cells. For the CRISPR/Cas9 system, PP7 aptamers were inserted into the tetraloop and stem-loop 2 of the sgRNA scaffold, the Cas9 nuclease mRNA sequence was flanked by MS2 aptamers on the RNA transfer plasmid, as detailed in supplementary Fig. S11, and the pLF-GagPol MA_MCP.ZF_PCP packaging plasmid was used for transfection. All newly generated constructs were verified by restriction enzyme digestion and sequencing. The primer sequences and additional details are available upon request. Production, concentration, and purification of LentiFlash® particles were done as described for the integrative viral particles.

The functionality of LentiFlash® particles carrying multiple RNA molecules encapsidated using a bacteriophage chimeric system, regardless of the position of the bacteriophage coat protein in the gag gene, was demonstrated and patented in 2015 [51]. We evaluated various designs to determine the best localization of the MS2 coat protein in the gag gene. We found that the maximum amount of RNAs is packaged into LentiFlash® particles when the MCP is inserted in the matrix gene. We did not observe any significant decrease of the p24 titers of LentiFlash® batches containing the MCP inserted into MA compared with constructs in which the MCP was inserted in the NC. The p24 titers of these batches remained high, and these particles effectively led to high and rapid level of expression of the gene of interest. Following the first LentiFlash® patent, the functionality of LentiFlash® particles to transfer the CRISPR/Cas9 system was then demonstrated and patented in 2016 [52]. We performed many additional experiments with this MCP-MA/PCP-NC optimized helper plasmid to confirm the best editing efficiency of these particles in different cell types and/or by targeting various genes.

For quantification, the p24 core antigen was detected directly in the viral supernatant with a HIV-1 p24 ELISA kit (Perkin Elmer), as specified by the supplier. The viral titer (expressed in physical particles per ml) was calculated from the p24 amount, knowing that 1 pg of p24 corresponds to 10^4^ physical particles.

### hiPSC transduction

The day before transduction, hiPSC were plated as single cells (25000 cells/cm^2^) in 24- or 48-well plates coated with Geltrex matrix in E8 medium supplemented with 10 μM of Y-27632. Two hours before transduction, the medium was changed with fresh E8 supplemented with 10 μM Y-27632. Transduction was performed using integrative lentiviral particles or LentiFlash® particles in E8 medium supplemented with 10 μM Y-27632 and 8 μg/ml polybrene (Sigma Aldrich) for 16 to 18 h before changing the medium.

### Electroporation

For electroporation, 4 × 10^5^ PCD_02:30 iPSC were transfected with 52 pmol Cas9 protein and 60 pmol premixed Alt-R CRISPR-Cas9 tracrRNA and Alt-R CRISPR-Cas9 crRNA (IDT DNA Technologies) in Nucleofector Solution SF (Lonza) in a final volume of 25 μl. Cells were transfected with the Amaxa 4D-Nucleofector (Lonza) device using program CA-137 and then resuspended in with E8 medium supplemented with 10 μM Y-27632 for 24 h.

### Sanger sequencing and ICE analysis

Targeted loci were amplified using the appropriate primers (listed in Supplementary Table S2) and the OneTaq® DNA Polymerase (NEB) following the manufacturer’s instruction. After amplification confirmation by electrophoresis of an aliquot on 2% agarose gels, PCR products were purified using the QIAquick PCR Purification Kit (QIAGEN), as recommended by the manufacturer. Purified PCR products were analyzed by Sanger sequencing (Eurofins Genomics). Chromatograms were analyzed with the freely available FinchTV version 1.4.0 (Geospiza) and ICE (Synthego) tools [53].

### On-target next-generation amplicon sequencing

Genomic DNA was used as input for PCR (primers described in Table S2) using OneTaq polymerase (New England Biolabs). PCR product was purified using the QIAquick Gel Extraction Kit (Qiagen) according to the manufacturer’s instructions and subjected to paired-end read sequencing on an Illumina flow cell MiniSeq Mid-Output at Montpellier GenomiX. Genomic PhiX DNA was spiked-in to increase base diversity. Fastq files were analyzed using the CRISPResso2 software with default settings [54].

### Clonal line derivation and validation

Clonal hiPSC lines were obtained by plating transduced cells at low density in 6-well plates coated with Geltrex matrix (from 500 to 10000 cells/well) in E8 medium supplemented with 10 μM Y-27632. The medium was changed every day and Y-27632 was added for the first 2 days. After 6–10 days, clones were manually picked and transferred to a well of a 96-well plate coated with Geltrex matrix in E8 medium. Clones were amplified in E8 medium for 5 to 8 days and passaged as small clumps in two wells of 96-well plates (duplicate). One plate was kept in culture and the second was used for screening.

Screening was performed by extracting DNA with the DNA Extract All Reagents Kit (Thermo Fisher Scientific) or the QIAamp DNA Blood Mini Kit (QIAGEN) followed by HRMA using the LightCycler® 480 SYBR Green kit (Roche) and a standard qPCR program (95 °C for 10 min, followed by 40 cycles of 95 °C for 10 s, 60 °C for 15 s, and 72 °C for 15 s, with a final melting curve including a step of 95 °C for 5 s followed by an increase from 65 to 97 °C with a temperature ramping set to 0.11 °C/s) on a LightCycler 480 (Roche). The identified mutant clones were confirmed by Sanger sequencing as described above.

Newly established clonal lines were validated by (1) Sanger sequencing analysis of off-target sites containing up to three mismatches (primers are listed in Supplementary Table S2), (2) immunofluorescence analysis of OCT3/4, NANOG, SOX2, and SSEA4 expression (pluripotency markers) (antibodies are listed in Supplementary Table S3), and (3) genomic integrity analysis by ddPCR of the copy number variations of the 24 more frequent recurrent genetic abnormalities that cover more than 90% of all recurrent genetic abnormalities found in hPSC (provided as a service by Stem Genomics) [55].

### Copy number variation analysis by droplet digital PCR (ddPCR)

Copy number variation was evaluated in a duplex reaction in which the target gene (*GFP* or *CCDC40*-YGT) is amplified using a FAM-labeled probe (IDT DNA Technologies) and the reference gene *RPP30* with a HEX-labeled probe (IDT DNA Technologies), set at two copies. Reaction mixes contained 150 ng of purified genomic DNA, 1x ddPCR Supermix (Bio-Rad), 900 nM of each primer (two primers per assay), and 250 nM of each probe (sequences detail can be found in Supplementary Table S2). Reaction mixes were loaded into DG8 cartridges with 70 μl of droplet oil (Bio-Rad) per sample and the droplets generated using the QX200 Droplet Generator (Bio-Rad), according to the manufacturer’s instructions. Emulsions were transferred to a 96-well plate, and samples were amplified in a thermocycler (95 °C for 10 min, followed by 45 cycles of 95 °C for 30 s and 60 °C for 60 s, with a final elongation step of 98 °C for 10 min with temperature ramping set at 2 °C/s). The plate was then loaded on the QX200 Droplet Reader (Bio-Rad), and copy number was determined with the QuantaSoft software.

### Gene expression analysis by real-time quantitative PCR

For qPCR experiments, total RNA was extracted from cells using the RNeasy® Mini kit (QIAGEN) following the manufacturer’s instructions. Reverse transcription to complementary DNA (cDNA) was performed using the SuperScript™ First-Strand Synthesis System for RT-PCR (Invitrogen) following the manufacturer’s instructions. qPCR was performed using LightCycler® 480 SYBR Green I Master (Roche) in 384 plates, in 10 μl reactions, including 1x SYBR Green I Master, 0.5 μM of each primer, and the cDNA. All reactions were performed in triplicate. A negative control (RNAse/DNAse-free water) was included in each run. The qPCR assays were performed on a LightCycler 480 (Roche) using the following program: 95 °C for 10 min, followed by 40 cycles of 95 °C for 10 s, 60 °C for 15 s, and 72 °C for 15 s, with a final melting curve including a step of 95 °C for 5 s followed by an increase from 65 to 97 °C with a temperature ramping set to 0.11 °C/s.

Raw data (Ct values) were analyzed using the comparative Ct method. Gene expression data were calculated as relative to the expression of the *GAPDH* housekeeping gene. The comparative threshold cycle method (ΔΔCT) was used to quantify relative gene expression, and the obtained quantification was transformed to 2^-ΔΔCT^ values.

### Immunofluorescence

Cells were fixed in 4% paraformaldehyde at room temperature (RT) for 15 min and then permeabilized with PBS containing 0.5% Triton X-100 (Thermo Fisher Scientific) for 15 min. Cells were blocked with 10% donkey serum in PBS containing 1% BSA and 0.1% Triton X-100 at RT for 60 min. Primary and secondary antibodies were diluted (see Supplementary Table S3 for details) in 1% BSA/0.1% Triton X-100/PBS. Samples were incubated with primary antibodies at 4 °C overnight and with secondary antibodies at RT in the dark for 60 min. Nuclei were stained with DAPI (1:2500) for 2 min and then samples were mounted in ProLong Gold (Thermo Fisher Scientific) and stored in the dark at RT. A Zeiss microscope (LMS700) and the ZEN 3.1 (blue edition) software were used for image acquisition and analysis.

### Flow cytometry analysis

For CXCR4 staining, cells were harvested by dissociation to a single cell suspension with Versene. Cells were first incubated with Zombie violet (1:1000, Biolegend) at RT in the dark for 15 min to differentiate between live and dead cells. Cell pellets were then incubated with a PE-conjugated anti-CXCR4 antibody (mouse PE, 1:200, BD Biosciences) or its isotype control at 4 °C for 30 min (see supplemental Table S3 for details). For GFP or ZsGreen analysis, cells were harvested by dissociation to single cell suspension using Versene and directly used for flow cytometry analysis.

All cells were analyzed using a Gallios flow cytometer (Beckman Coulter) and the Kaluza Analysis software (Beckman Coulter).

### Lactate dehydrogenase cytotoxicity assay and cell counting

LDH cytotoxicity analysis was performed using the Cytotoxicity Detection Kit (LDH) (Roche) following the manufacturer’s instructions. Briefly, supernatants of transduced and control cells were harvested every day for 3 days. A cell lysate control was made using a well of not transduced cells lysed in 1% Triton X-100 solution. Fresh E8 medium constituted the blank control. Supernatants, blank, and lysis controls were centrifuged at 5000 rpm for 3 min to remove cells and cell debris. Then, supernatants (100 μl/each) were distributed in duplicate in a 96-well plate. One hundred microliters of reaction mixture containing the catalyst and the dye solution was added to each well, and the plate was incubated in the dark at RT for 15 min. Absorbance was measured at 492 nm using a Varioskan Flash reader (Thermo Fischer Scientific).

Cells were counted manually using a Malassez counting chamber after dissociation to single cells using Versene.

### Differentiation to definitive endoderm

hiPSC were differentiated to definitive endoderm as per Ahmed et al. [45]. Briefly, 16 to 18 h before differentiation induction, cells were plated as single cells at 80000 cells/cm^2^ in 24- or 6-well plates in E8 medium supplemented with 10 μM Y-27632. Differentiation was induced by replacing the E8 medium with RPMI 1640 (GIBCO) supplemented with B27 without vitamin A (GIBCO), 100 ng/ml activin A (Peprotech), 3 mM CHIR-99021 (Tocris), and 10 μM Y-27632. After 24 h, the medium was changed to RPMI 1640 supplemented with B27 without vitamin A, 100 ng/ml activin A, and 250 nM LDN-193189 (Miltenyi biotec). Cells were then incubated for another 24 h to obtain definitive endoderm.

### Differentiation to neural progenitors and transduction

hiPSC were differentiated to neural progenitors using the STEMdiff^TM^ SMADi Neural Induction kit (STEMCELL), following the manufacturer’s instructions. Briefly, differentiation was induced by plating hiPSC as single cells at 200,000 cells/cm^2^ in 6-well plates coated with Geltrex matrix in STEMdiff^TM^ Neural Induction medium supplemented with SMADi and 10 μM Y-27632. After 24 h, the medium was changed to fresh STEMdiff^TM^ Neural Induction medium supplemented with SMADi. Then, the medium was changed daily for 4 to 5 days before cell passaging. For this, cells were dissociated to single cell using Versene followed by plating 150,000 cells/cm^2^ in 6-well plates coated with Geltrex matrix in STEMdiff^TM^ Neural Induction medium supplemented with SMADi and 10 μM Y-27632.

The day prior to transduction, neural progenitors were dissociated to single cell using Versene and 125,000 cells/cm^2^ were plated in 48-well plates coated with Geltrex matrix in STEMdiff^TM^ Neural Induction medium supplemented with SMADi and 10 μM Y-27632. Two hours before transduction, the medium was changed to fresh STEMdiff^TM^ Neural Induction medium supplemented with SMADi and 10 μM Y-27632. Transduction was performed using LentiFlash® particles in STEMdiff^TM^ Neural Induction medium supplemented with SMADi, 10 μM Y-27632, and 8 μg/ml polybrene for 16 to 18 h before changing the medium.

### Statistical analysis

Data are presented as the mean ± SEM. Differences were evaluated using the two-way ANOVA, Dunnett test. Statistical analyses were performed with GraphPad Prism. A value of *P* ≤ 0.05 was considered to be significant.

## Supplementary Information

**Additional file 1: Figs. S1-S11.** **Supplementary Figure S1.** Transduction of the HY03 hiPSC line with LF-ZsGreen particles allows transient ZsGreen expression and does not affect pluripotency. **Supplementary Figure S2.** LF-ZsGreen particles allow the highly efficient delivery of RNA in different hiPSC lines. **Supplementary Figure S3.** Characterization of the HY03-GFP non-clonal reporter hiPSC line. **Supplementary Figure S4.** NGS analysis of DNAH5 indel size distribution. **Supplementary Figure S5.** LentiFlash® particle-based transduction of the CRISPR/Cas9 system to target the GFP fluorescent reporter sequence in a HCT116-GFP cell line that contains one GFP copy per cell. **Supplementary Figure S6.** Characterization of hiPSC clones in which *DNAH5* or *MCIDAS* was knocked out by CRISPR/Cas9 gene editing using the LentiFlash® system. **Supplementary Figure S7.** LentiFlash® particle-based transduction of the CRISPR/Cas9 components to target specific genes results in high indel formation in different hiPSC lines. **Supplementary Figure S8.** Interallelic gene conversion following iPSC electroporation and following LentiFlash® transduction of neural progenitors obtained from the PCD_02:30 hiPSC line results. **Supplementary Figure S9.** Characterization of hiPSC clonal lines harboring *DNAH5* heterozygous mutations. **Supplementary Figure S10.** Specifically targeting the wild-type allele in hiPSC clones harboring a *DNAH5* heterozygous mutation results in interallelic gene conversion. **Supplementary Figure S11.** Schematic representation of the expression cassettes carried by the plasmids used to produce the integrative lentiviral vector (ILV) that expresses the GFP reporter and of the expression cassettes carried by the plasmids used to produce non-integrative lentiviral particles (LentiFlash®) that express the ZsGreen reporter or the CRISPR/Cas9 systems. **Supplementary Tables S1-3.** **Supplementary Table S1.** sgRNA sequences. **Supplementary Table S2.** PCR primers. **Supplementary Table S3.** antibodies

## Data Availability

The datasets analyzed in the current study are available from the corresponding author.

## Abbreviations

Cas9: CRISPR-associated protein 9
cDNA: Complementary DNA
CCDC40: Coiled-coil domain containing 40
COPD: Chronic obstructive pulmonary disease
CRISPR: Clustered regularly interspaced short palindromic repeats
CXCR4: C-X-C chemokine receptor type 4
ddPCR: Droplet digital PCR
DSB: Double-strand breaks
hiPSC: Human induced pluripotent stem cells
hPSC: Human pluripotent stem cells
HRMA: High-resolution melting analysis
ICE: Interference of CRISPR edits
IDRV: Integration-deficient retroviral vector
ILV: Integrative lentiviral vector
LDH: Lactate dehydrogenase
LF-ZsGreen: LentiFlash® particles containing the fluorescent reporter ZsGreen mRNA
LVLP: Lentivirus-like particles
MCIDAS: Multiciliate differentiation and DNA synthesis associated cell cycle protein
MFI: Mean fluorescent intensity
MMEJ: Micro-homology end-joining
NHEJ: Non-homologous end-joining
NT: Not transduced
OT: Off-target
PCD: Primary ciliary dyskinesia
PFV: Prototype foamy virus
qPCR: Quantitative PCR
sgRNA: Single guide RNA
SIN: Self-inactivating
RRE: Rev-responsive element
RT: Room temperature
TRAC: T cell receptor alpha constant
TU: Transduction unit
VSV-G: Vesicular stomatitis virus envelope glycoprotein
WPRE: Woodchuck hepatitis virus post-transcriptional regulatory element
WT: Wild type allele
