# Supplementary material for: CRISPR/Cas9-mediated gene knockout and interallelic gene conversion in human induced pluripotent stem cells using non-integrative bacteriophage-chimeric retrovirus-like particles
Source: BMC Biol. 2022 Jan 7;20:8. doi: 10.1186/s12915-021-01214-x (PMC8742436; doi:10.1186/s12915-021-01214-x)
Supplement: Supplementary file 1 — Additional file 1: Figs. S1-S11. Supplementary Figure S1. Transduction of the HY03 hiPSC line with LF-ZsGreen particles allows transient ZsGreen expression and does not affect pluripotency. Supplementary Figure S2. LF-ZsGreen particles allow the highly efficient delivery of RNA in different hiPSC lines. Supplementary Figure S3. Characterization of the HY03-GFP non-clonal reporter hiPSC line. Supplementary Figure S4. NGS analysis of DNAH5 indel size distribution. Supplementary Figure S5. LentiFlash® particle-based transduction of the CRISPR/Cas9 system to target the GFP fluorescent reporter sequence in a HCT116-GFP cell line that contains one GFP copy per cell. Supplementary Figure S6. Characterization of hiPSC clones in which DNAH5 or MCIDAS was knocked out by CRISPR/Cas9 gene editing using the LentiFlash® system. Supplementary Figure S7. LentiFlash® particle-based transduction of the CRISPR/Cas9 components to target specific genes results in high indel formation in different hiPSC lines. Supplementary Figure S8. Interallelic gene conversion following iPSC electroporation and following LentiFlash® transduction of neural progenitors obtained from the PCD_02:30 hiPSC line results. Supplementary Figure S9. Characterization of hiPSC clonal lines harboring DNAH5 heterozygous mutations. Supplementary Figure S10. Specifically targeting the wild-type allele in hiPSC clones harboring a DNAH5 heterozygous mutation results in interallelic gene conversion. Supplementary Figure S11. Schematic representation of the expression cassettes carried by the plasmids used to produce the integrative lentiviral vector (ILV) that expresses the GFP reporter and of the expression cassettes carried by the plasmids used to produce non-integrative lentiviral particles (LentiFlash®) that express the ZsGreen reporter or the CRISPR/Cas9 systems. Supplementary Tables S1-3. Supplementary Table S1. sgRNA sequences. Supplementary Table S2. PCR primers. Supplementary Table S3. antibodies [file 12915_2021_1214_MOESM1_ESM.pdf]

Efficient CRISPR/Cas9-mediated gene knockout and interallelic gene conversion in human induced pluripotent stem cells using non-integrative bacteriophage-chimeric retrovirus-like particles

Joffrey Mianné1, Amel Nasri1, Chloé Nguyen Van1, Chloé Bourguignon1, Mathieu Fieldès1, Engi Ahmed1, Christine Duthoit2, Nicolas Martin2, Hugues Parrinello3,4, Anaïs Louis3,4, Alexandra Iché2, Régis Gayon2, Florine Samain2, Lucille Lamouroux2, Pascale Bouillé2, Arnaud Bourdin5, Said Assou1, John De Vos1,6*

Supplementary Materials

Supplementary figures

Legend to Supplementary Figures

Supplementary Figure S1: Transduction of the HY03 hiPSC line with LF-ZsGreen particles allows transient ZsGreen expression and does not affect pluripotency. **a** Time course analysis of ZsGreen expression in HY03 cells transduced with LF-ZsGreen particles at 0.5, 2 or 5 pg p24/cell. Fluorescent microscopy analysis of cells during the first 6 days post-transduction. **b** Fold change of ZsGreen fluorescence intensity measured by flow cytometry analysis during 10 days post-transduction (NT, not-transduced cells = 1). **c** Immunofluorescence analysis of pluripotency markers (OCT3/4, SSEA4, NANOG and SOX2) in HY03 cells transduced with 2 pg p24/cell LF-ZsGreen particles (at passage 2 post-transduction). 488 and 555, Alexa-Fluor 488 and 555 negative controls. **d** Characterization of definitive endoderm cells obtained from the directed differentiation of HY03 hiPSC cells transduced with 2 pg p24/cell of LF-ZsGreen particles. Left: CXCR4 flow cytometry analysis. Right: Immunofluorescence microscopy analysis of FOXA2 and SOX17 expression. PE, phycoerythrin staining.

Supplementary Figure S2: LF-ZsGreen particles allow the highly efficient delivery of RNA in different hiPSC lines. **a** Fluorescent microscopy analysis of the iCOPD9_B27 (left) and PCD_02:30 (right) hiPSC lines at 48h post-transduction with LF-ZsGreen particles at 0.5, 2 or 5 pg p24/cell. **b** Transduction efficacy in iCOPD9_B27 and PCD_02:30 cells measured by flow cytometry quantification of ZsGreen-positive cells at 48h post-transduction. NT, not-transduced cells. **c** Mean fluorescent intensity (MFI) fold change in iCOPD9_B27 and PCD_02:30 cells assessed by cytometry analysis at 48h post-transduction (NT, not-transduced cells = 1). **d** Immunofluorescence analysis of pluripotency markers (OCT3/4, SSEA4, NANOG and SOX2) in iCOPD9_B27 (left) and PCD_02:30 (right) cells transduced with 2 pg p24/cell of LF-ZsGreen particles (passage 2 after transduction). 488 and 555, Alexa-Fluor 488 and 555 negative controls.

Supplementary Figure S3: Characterization of the HY03-GFP non-clonal reporter hiPSC line. **a** Fluorescent microscopy analysis of GFP expression. **b** GFP allele copy number (average = 2.42) measured by ddPCR. **c** Immunofluorescence analysis of pluripotency markers (OCT3/4, SSEA4, NANOG and SOX2). 488 and 555, Alexa-Fluor 488 and 555 negative controls. **d** Genomic integrity analysis by ddPCR of the 24 most recurrent loci found aneuploid in hiPSC.

Supplementary Figure S4: NGS analysis of DNAH5 indel size distribution. CRISPResso2 analysis of DNAH5 indel size distribution by NGS following LentiFlash® transduction of HY03 iPSC.

Supplementary Figure S5: LentiFlash® particle-based transduction of the CRISPR/Cas9 system to target the GFP fluorescent reporter sequence in a HCT116-GFP cell line that contains one GFP copy per cell. Data were obtained by flow cytometry on day 7 post-transduction. NT: non-transduced cells. LentiFlash® particles were produced with (i) an expression plasmid containing only Cas9-MS2, (ii) two expression plasmids containing sgRNAGFP-PP7 and Cas9 without the MS2 aptamers, respectively, (iii) two expression plasmids containing sgRNAGFP-PP7 or Cas9-MS2, respectively, and (iv) sgRNAGFP-PP7 and Cas9-MS2 in a single plasmid.

Supplementary Figure S6: Characterization of hiPSC clones in which *DNAH5* or *MCIDAS* was knocked out by CRISPR/Cas9 gene editing using the LentiFlash® system. **a** Nucleotide sequence analysis of the *DNAH5* and *MCIDAS* loci in the DNAH5_A4 (left) and MCIDAS_E1 (right) knock-out clones, respectively. The DNAH5_A4 line harbors a heterozygote composite mutation with one allele carrying a combination of 4-nucleotide deletion and 2-nucleotide insertion (Del4ins2 allele), and a second allele a 7-nucleotide deletion (Del7 allele). The MCIDAS_E1 line harbors a homozygote mutation with 1 nucleotide inserted at the targeted locus (Ins1 alleles). *indicates the nucleotide insertion. **b** Immunofluorescence analysis of pluripotency markers (OCT3/4, SSEA4, NANOG and SOX2) in DNAH5_A4 (left) and MCIDAS_E1 (right) cells. 488 and 555, Alexa-Fluor 488 and 555 negative controls. **c** Analysis of potential off-target (OT) sites containing up to 3 mismatches by Sanger sequencing of DNAH5_A4 (top) and MCIDAS_E1 cells (bottom). MMs, mismatches. **d** Genomic integrity analysis by ddPCR of the 24 most recurrent loci found aneuploid in hiPSC. **e** Cas9 genotyping PCR. First lane : no template. Second lane : positive control (pX458 plasmid).

Supplementary Figure S7: LentiFlash® particle-based transduction of the CRISPR/Cas9 components to target specific genes results in high indel formation in different hiPSC lines. Indel rate at four loci (*MCIDAS*, *DNAH5*, *TRAC* and *CXCR4*) using optimized particle doses (0.5, 0.5, 2 and 7.5 pg p24/cell for *MCIDAS*, *DNAH5*, *TRAC* and *CXCR4* respectively) in the HY03, iCOPD9_B27 and PCD_02:30 hiPSC lines at day 3 post-transduction. Data were obtained by ICE decomposition analysis after Sanger sequencing of the targeted loci.

Supplementary Figure S8: Interallelic gene conversion following iPSC electroporation and following LentiFlash® transduction of neural progenitors obtained from the PCD_02:30 hiPSC line results. **a** Interallelic gene conversion after electroporation of PCD_02:30 iPSC with CRISPR/Cas9 RNPs targeting the Δ-2nt allele. Allelic composition at the targeted locus by ICE analysis. NT: no transduction **b** Immunofluorescence analysis of neuronal markers (human nestin and PAX6) in neural progenitors obtained from the differentiation of PCD_02:30 cells after 12 days of induction. 555, Alexa-Fluor 555 negative control. **c** Targeting of the Δ-2nt allele by transducing PCD_02:30 neural progenitor cells with LF-CRISPR/Cas9-CCDC40-YGT particles at 0.5, 2 and 5 pg p24/cell results in dose-dependent interallelic gene conversion. Left: Allelic composition at the targeted locus. Right: Representative Sanger sequencing chromatograms. ICE, Inference of CRISPR Edits; WT, wild-type; NT, not-transduced.

Supplementary Figure S9: Characterization of hiPSC clonal lines harboring *DNAH5* heterozygous mutations. **a** Nucleotide sequence composition for the DNAH5_A6, DNAH5_C3 and DNAH5_B5 clones harboring *DNAH5* heterozygous mutations obtained by LF-CRISPR/Cas9-DNAH5 particle-based transduction. Top: Sanger sequencing chromatograms. Bottom: nucleotide composition at the targeted locus. In DNAH5_A6 cells, the mutant allele harbors a 7-nucleotide deletion (Del7 allele [Δ-7nt]), DNAH5_C3 cells a 1-nucleotide insertion (Ins1 allele [Δ+1nt]) and DNAH5_B5 a 1-nucleotide deletion associated with a 2-nucleotide insertion (Del1/Ins2 allele [Δ-1/+2nt]). **b** Immunofluorescence analysis of pluripotency markers (OCT3/4, SSEA4, NANOG and SOX2) in DNAH5_A6 (top left), DNAH5_C3 (top right) and DNAH5_B5 (bottom) cells. 488 and 555, Alexa-Fluor 488 and 555 negative controls. **c** Analysis of potential OT sites containing up to 3 mismatches by Sanger sequencing of DNAH5_A6, DNAH5_C3 and DNAH5_B5 cells. MMs, mismatches. **d** Genomic integrity analysis by ddPCR

analysis of the 24 most recurrent loci found aneuploid in hiPSC DNAH5_A6 (top left), DNAH5_C3 (top right) and DNAH5_B5 (bottom).

Supplementary Figure S10: Specifically targeting the wild-type allele in hiPSC clones harboring a *DNAH5* heterozygous mutation results in interallelic gene conversion. Targeting the WT allele in DNAH5_A6, DNAH5_C3 and DNAH5_B5 cells by transduction of LF-CRISPR/Cas9-DNAH5 particles at 0.5 pg p24/cell resulted in interallelic gene conversion. **a** ICE analysis results before and after transduction in hiPSC DNAH5_A6 (top left), DNAH5_C3 (top right) and DNAH5_B5 (bottom). **b** Sanger sequencing chromatograms showing the allelic composition at the targeted locus in the three cell lines before and after LF-CRISPR/Cas9-DNAH5 particles transduction. WT, wild-type.

Supplementary Figure S11: (A) Schematic representation of the expression cassettes carried by the plasmids used to produce the integrative lentiviral vector (ILV) that expresses the GFP reporter. pLV-GFP: GFP expression cassette encoded by the HIV-1 self-inactivating (SIN) transfer plasmid and driven by the short EF1 alpha promoter (EFS). Indicated are the RSV-U3 5' LTR (chimeric long-terminal repeat composed of Rous Sarcoma Virus U3 promoter, HIV-1 R and HIV-1 U5), rev-responsive element (RRE), woodchuck hepatitis virus post-transcriptional regulatory element (WPRE) and SIN HIV-1 3' LTR (ΔU3, R, U5); pLV-GagPol (wt): standard HIV-1 trans-complementation packaging sequence expressed under the control of the CMV promoter; pVSVg: vesicular stomatitis virus envelope glycoprotein sequence expressed under the control of the CMV promoter. (B) Schematic representation of the expression cassettes carried by the plasmids used to produce non-integrative lentiviral particles (LentiFlash®) that express the ZsGreen reporter (top) or the CRISPR/Cas9 systems (bottom). pLF-ZsGreen is a construct with a PP7-driven RNA packaging sequence carrying the ZsGreen gene expressed under the EF1 alpha promoter with two copies of the 25-nt RNA stem-loop from the PP7 bacteriophage (PP7_2X) inserted in the 3' untranslated region. pLF-GagPol ZF_PCP is the packaging construct that contains the PP7 coat protein at the place of the second zinc finger (ZF2) of the NC. pLF_CRISPR/Cas9 is a construct with heterologous PP7/MS2-driven RNA packaging sequences carrying: i) a single-guide RNA (sgRNA) sequence under the control of the U6 promoter with two copies of the 25-nt RNA stem-loop from the PP7 bacteriophage (PP7_2X) inserted in the tetraloop and stem-loop 2 of the sgRNA scaffold, respectively, and ii) the SpCas9 gene expressed under the control of the EF1 promoter with 12 copies of the 19-nt RNA stem-loop from MS2 bacteriophage (MS2_12X) inserted in the 3' untranslated region. pLF-GagPol MA_MCP.ZF_PCP is the packaging construct containing the MS2 coat protein inserted between the 127th and 128th amino acid of the MA protein and PP7 coat protein at the place of the ZF2 of the NC. CMV: human cytomegalovirus immediate early enhancer and promoter; MA: matrix; CA: capsid; NC: nucleocapsid; POL: polymerase; Ins2-pA: rat insulin-2 polyadenylation signal; HBB intron: internally truncated intron from human β-globin; HBB-pA: human β-globin polyadenylation signal; BGH-pA: Bovine growth hormone polyadenylation signal.

Supplementary Figure S1

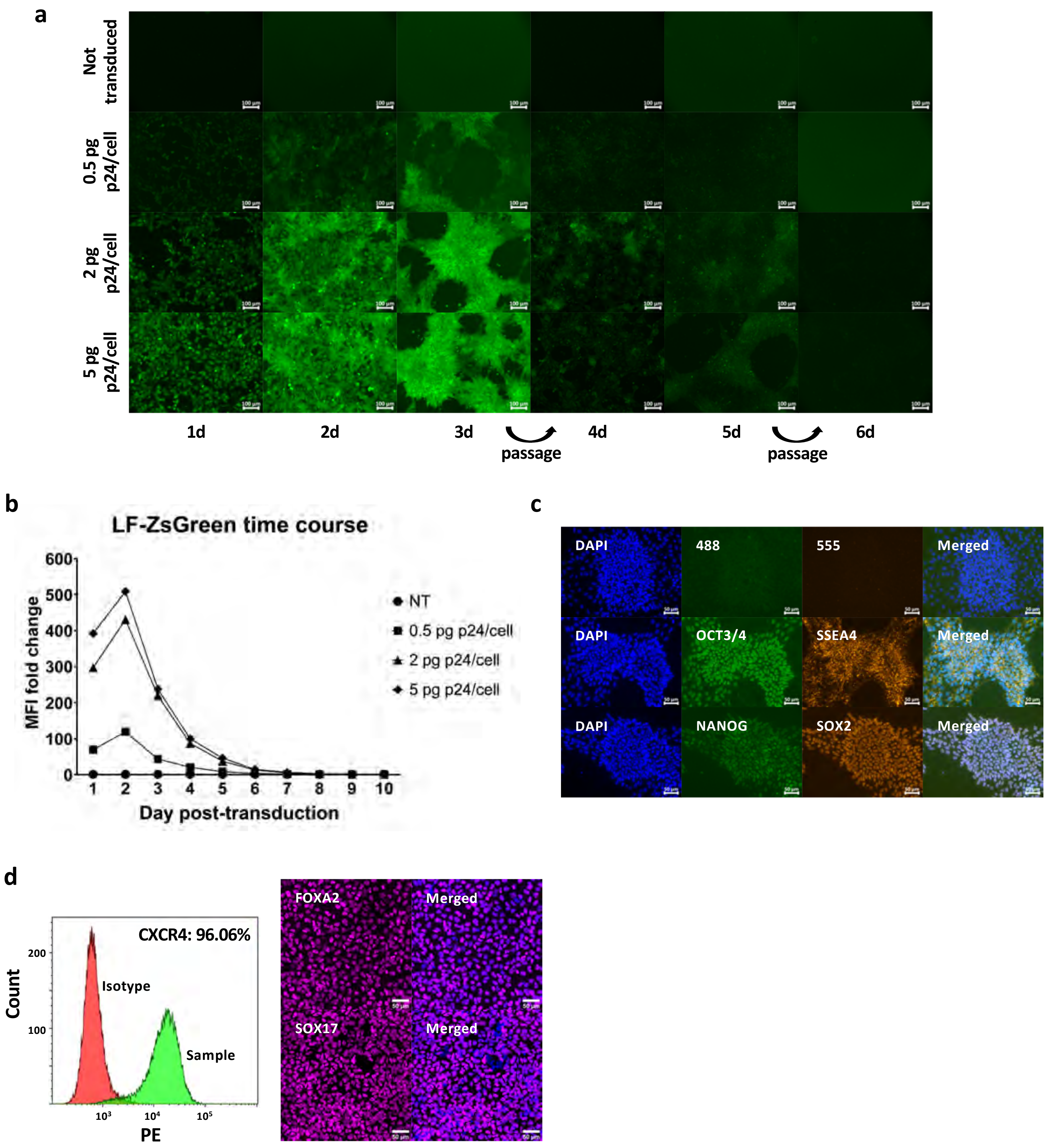

Supplementary Figure S2

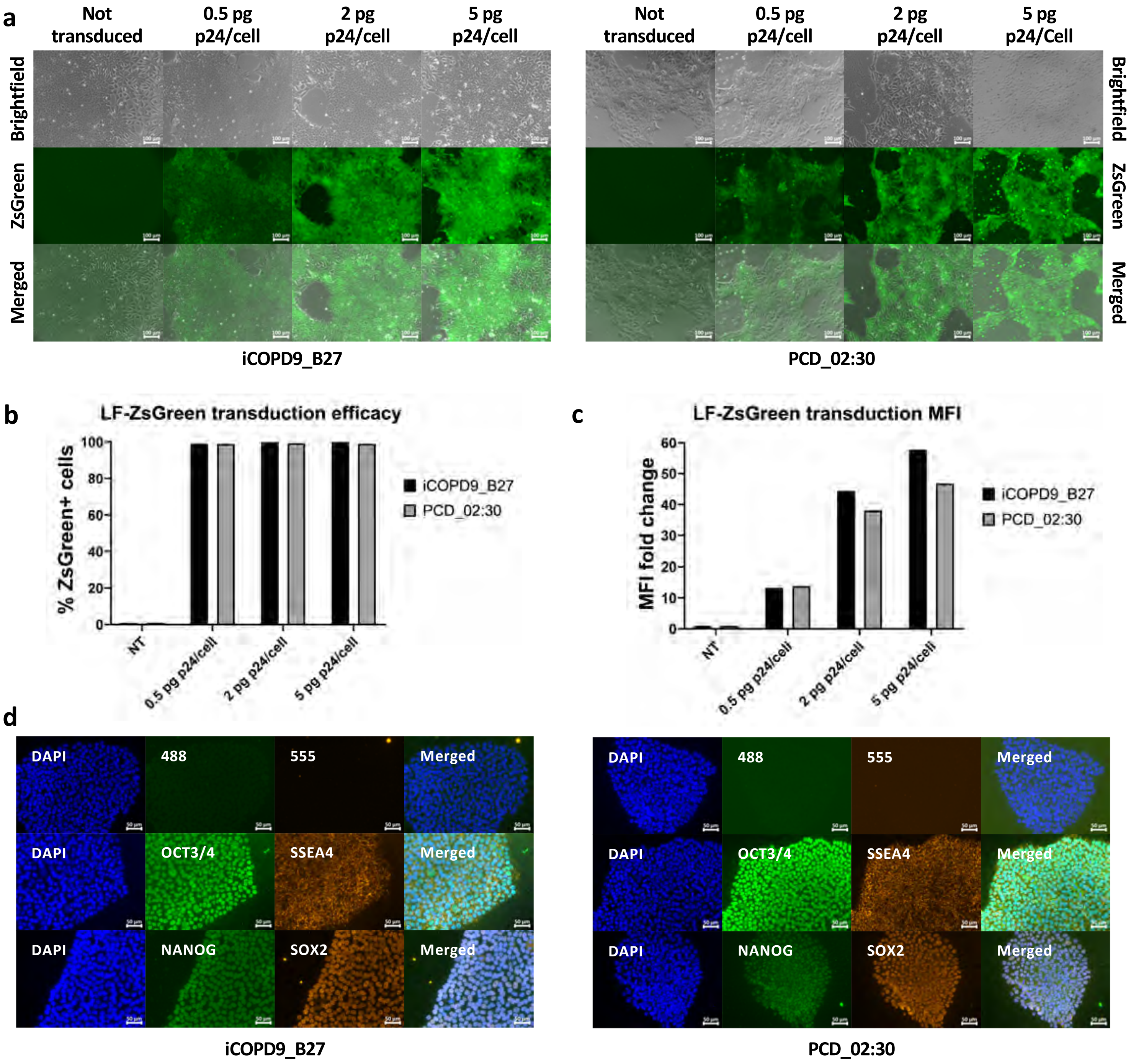

Supplementary Figure S3

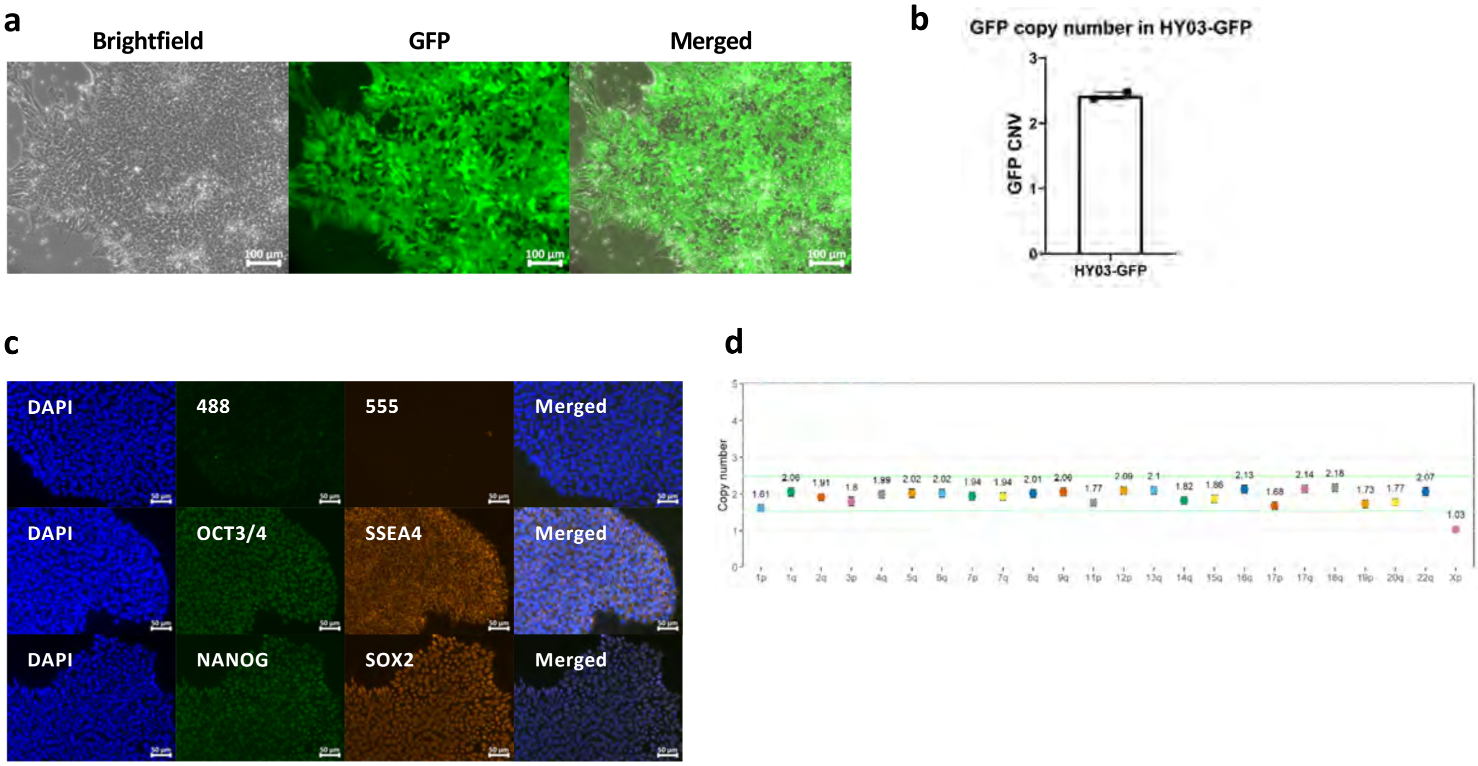

Supplementary Figure S4

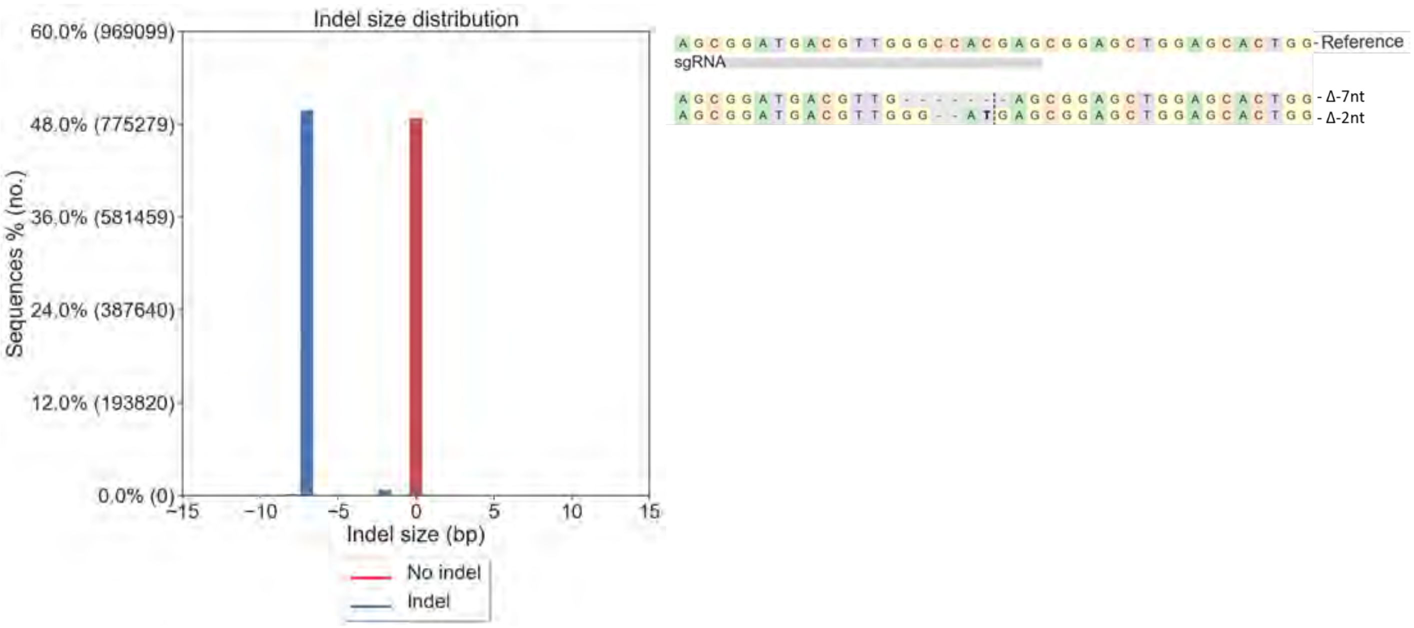

Supplementary Figure S5

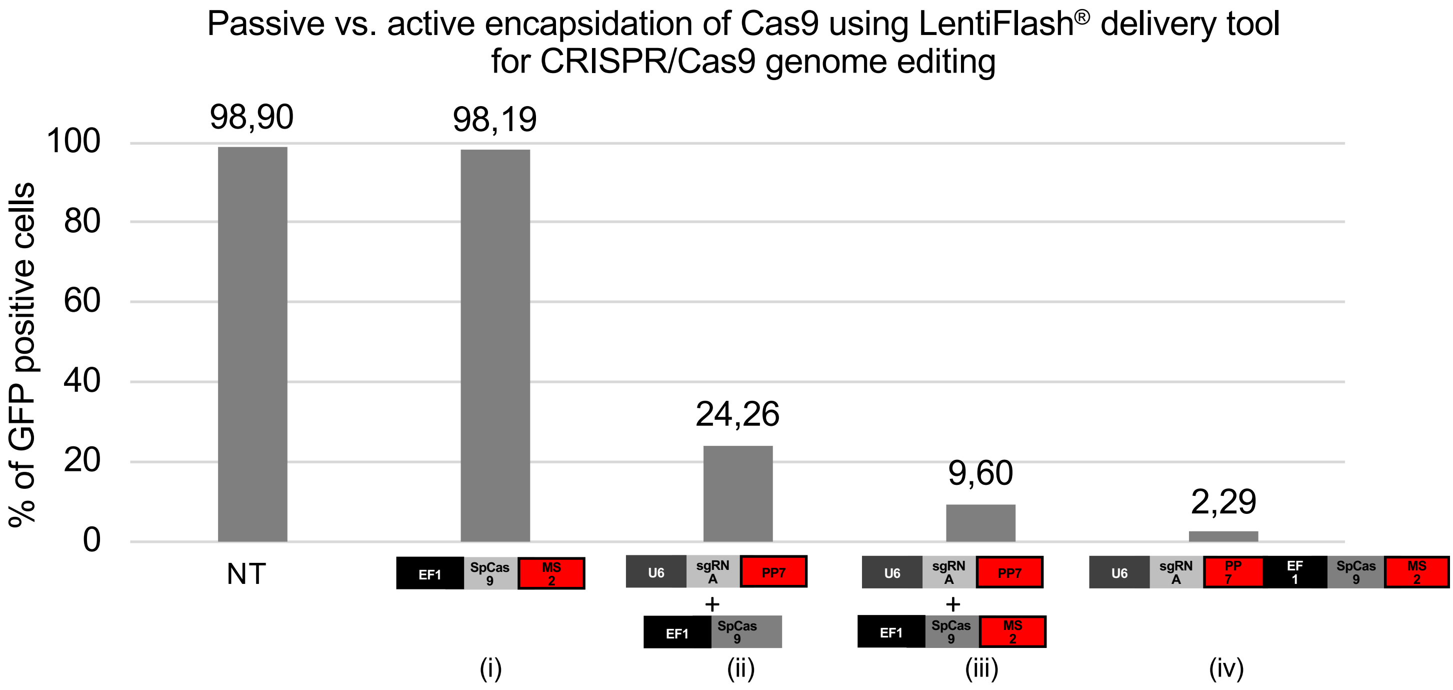

Supplementary Figure S6

a

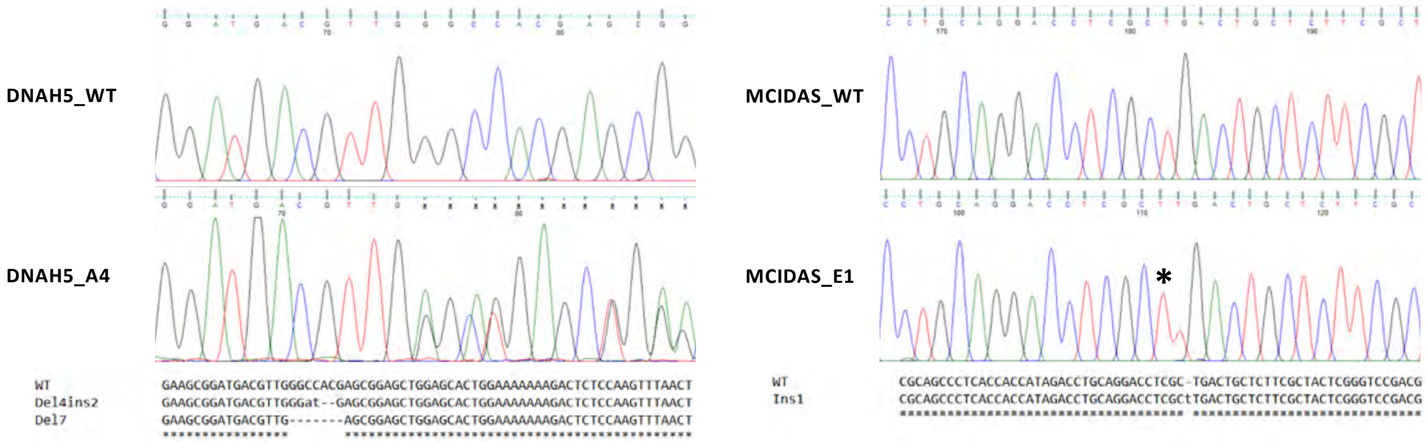

b

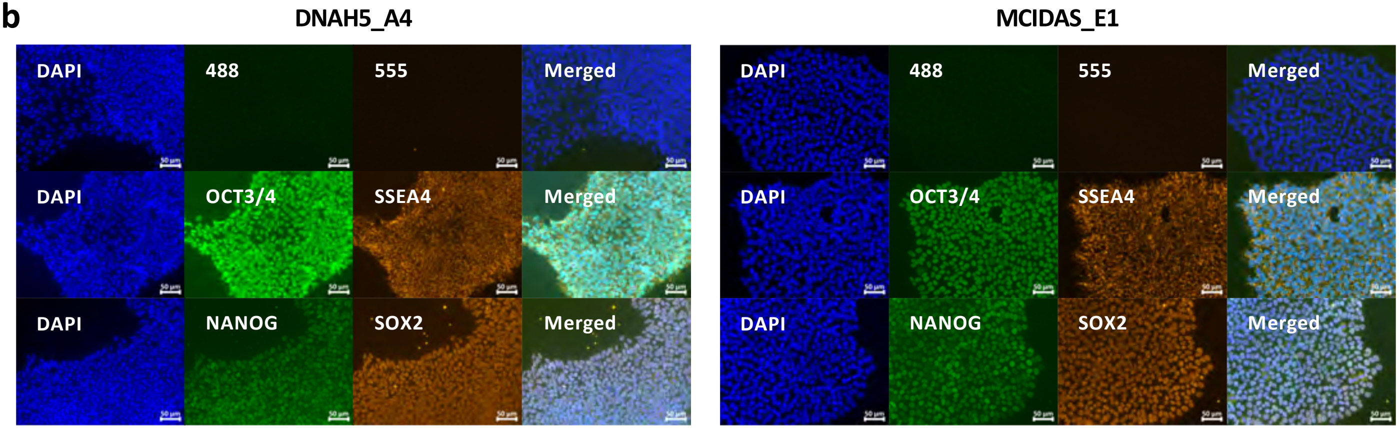

c

DNAH5_A4

Sequence	Location	Sequence	MMs	Type	A4
On-target	5:13919295-13919317	GGATGACGTTGGGCCACGAG CGG	0	Exonic	/
OT_1	13:76474926-76474948	GGATAACCTTGGGCTACGAG GGG	3	Intergenic	WT
OT_2	17:76527465-76527487	GGATGAGGATGGGCCAGGAG GGG	3	Exonic	WT
OT_3	13:111514874-111514896	GGATGACGCTGGGCCCCGTG TGG	3	Intergenic	WT

MCIDAS_E1

Sequence	Location	Sequence	MMs	Type	E1
On-target	5:55226620-55226642	GTAGCGAAGAGCAGTCAGCG AGG	0	Exonic	/
OT1	6:167932507-167932529	AAAGCGAATAGCAGTCAGCG TGG	3	Intronic	WT
OT2	22:41256116-41256138	GGAGGGAAGAGCAGTCAGCC GGG	3	Intronic	WT
OT3	19:41998579-41998598	GAAGCGGAAAGCAGTCAGCG AAG	3	Exonic	WT
OT4	8:135020309-135020328	GGAACAAAGAGCAGTCAGCG AGA	3	Intergenic	WT
OT5	7:73443058-73443080	GCAGCAAAGAGCAGGCAGCG GGG	3	Intronic	WT
OT6	X:142104307-142104326	GTCTCGAAGAGCAGTCAGAG AGA	3	Intergenic	WT

d

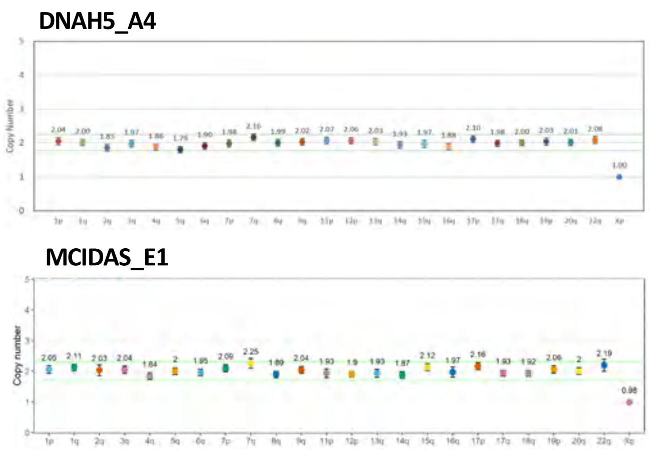

e

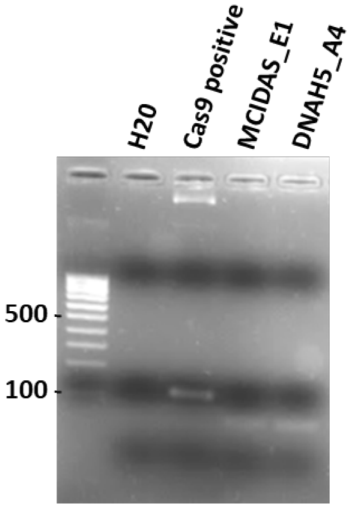

Supplementary Figure S7

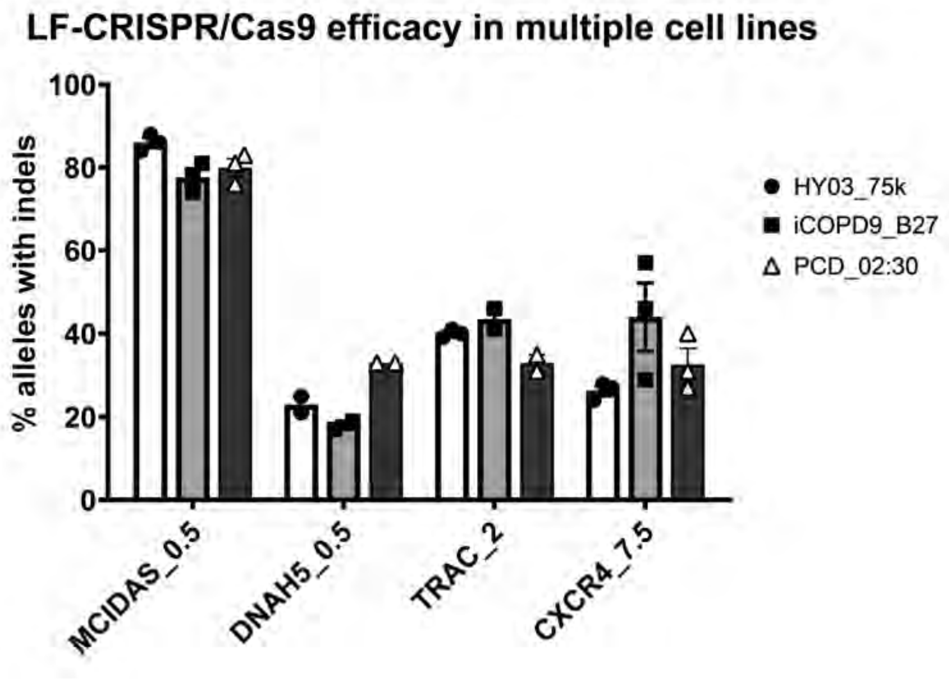

Supplementary Figure S8

a

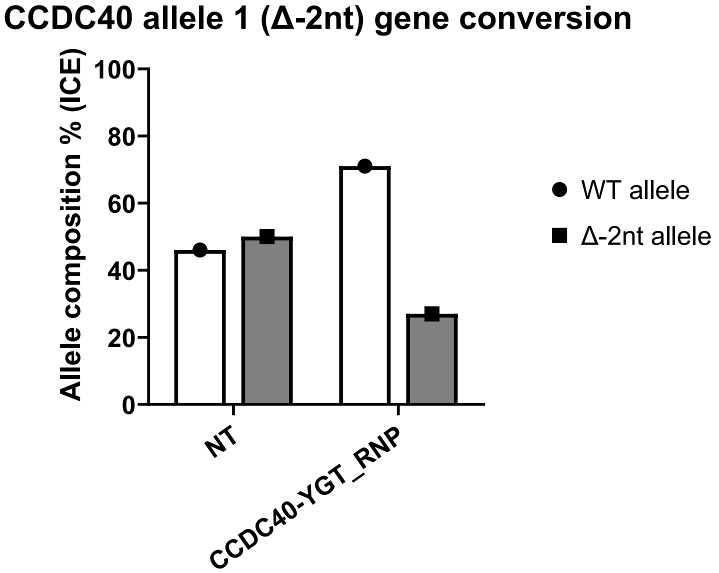

b

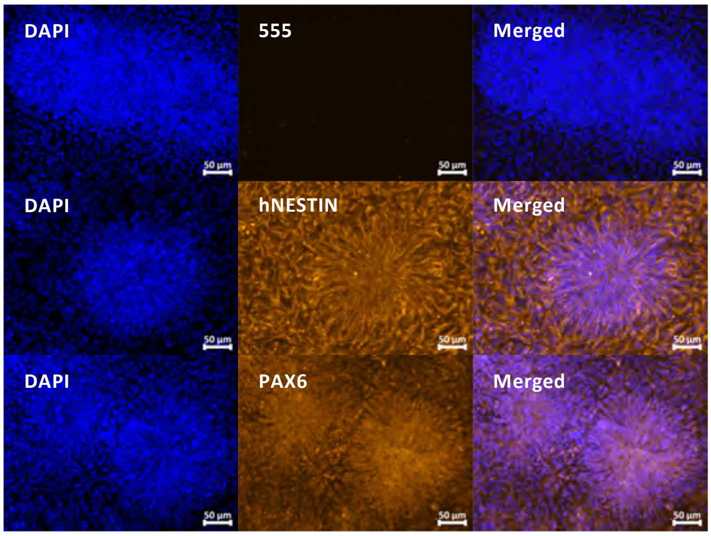

c

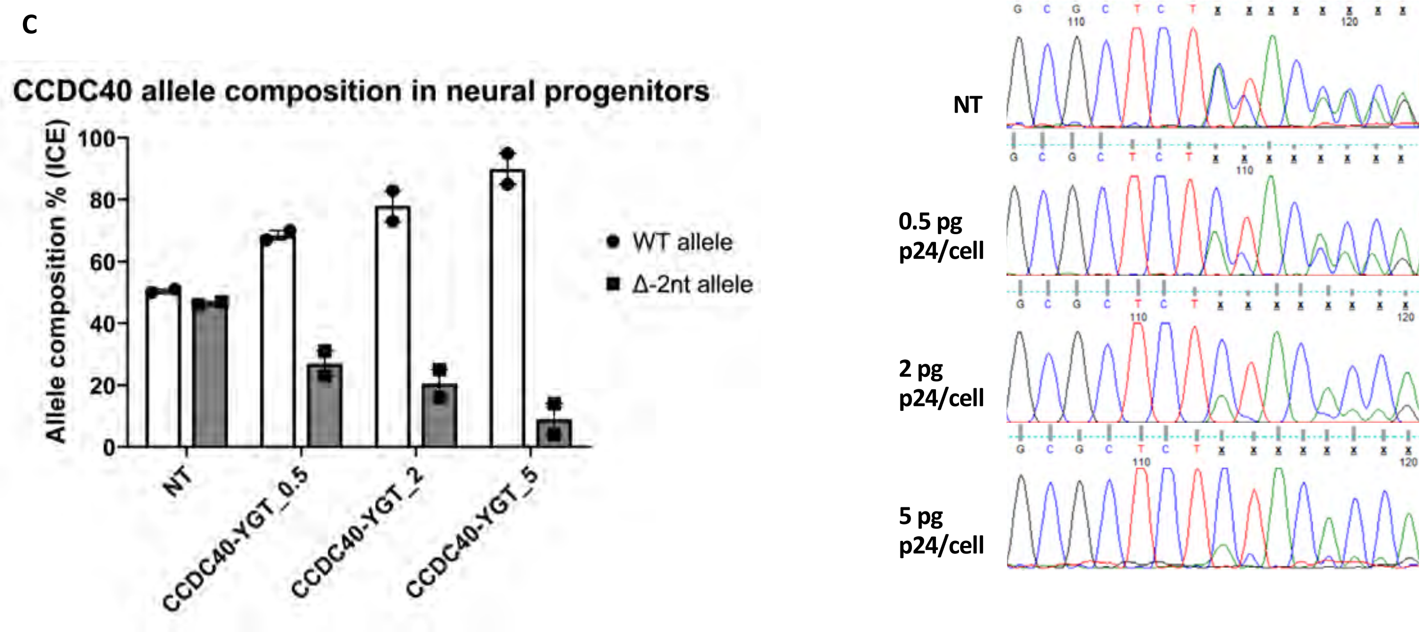

Supplementary Figure S9

a

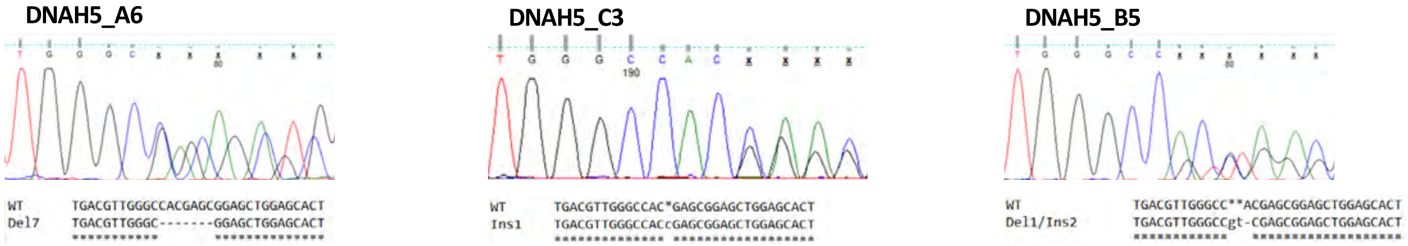

b

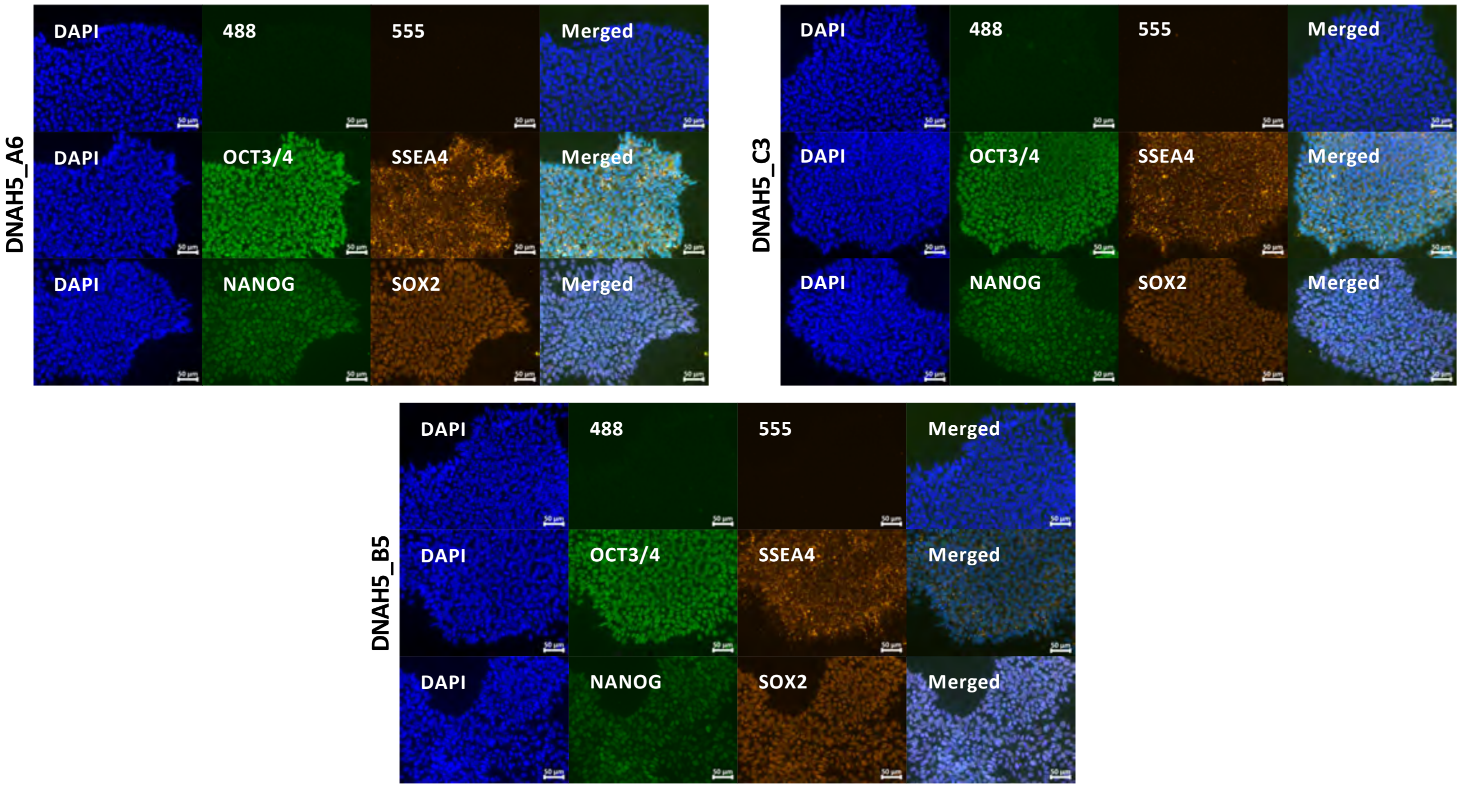

c

Sequence	Location	Sequence	MMs	Type	A6	C3	B5
On-target	5:13919295-13919317	GGATGACGTTGGGCCACGAG CGG	0	Exonic	Het	Het	Het
OT_1	13:76474926-76474948	GGAT**A**AC**C**TTGGGC**T**ACGAG GGG	3	Intergenic	WT	WT	WT
OT_2	17:76527465-76527487	GGATGA**G**G**A**TGGGCCA**G**GAG GGG	3	Exonic	WT	WT	WT
OT_3	13:111514874-111514896	GGATGACG**C**TGGGCC**C**CG**T**G TGG	3	Intergenic	WT	WT	WT

d

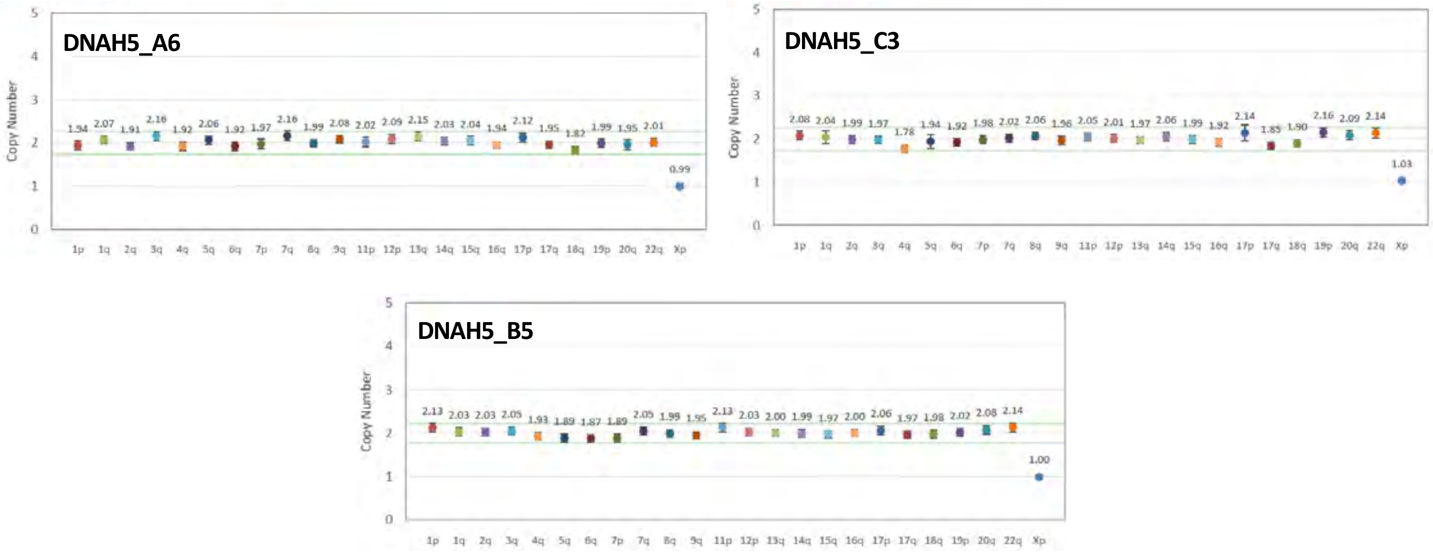

Supplementary Figure S10

a

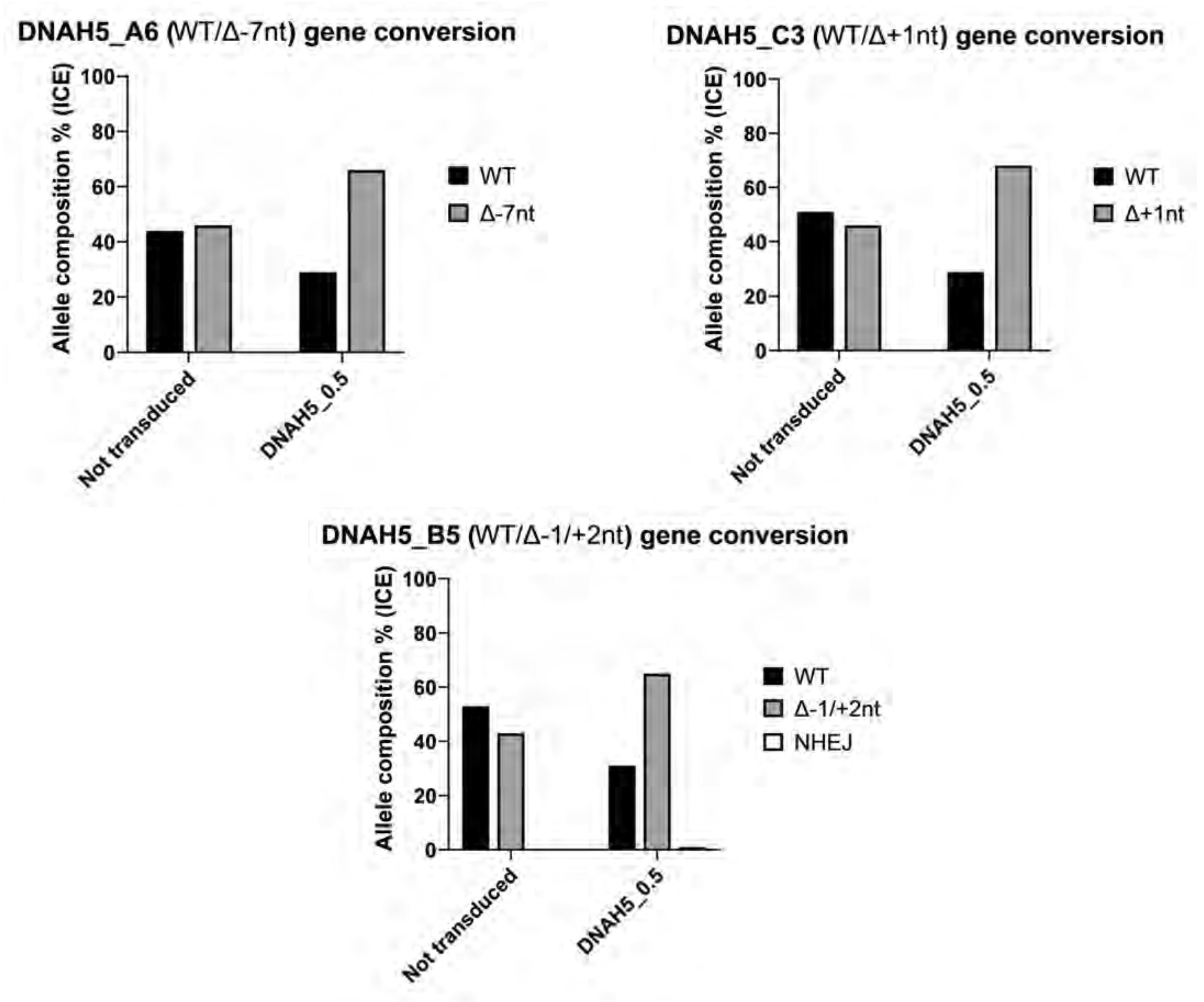

b

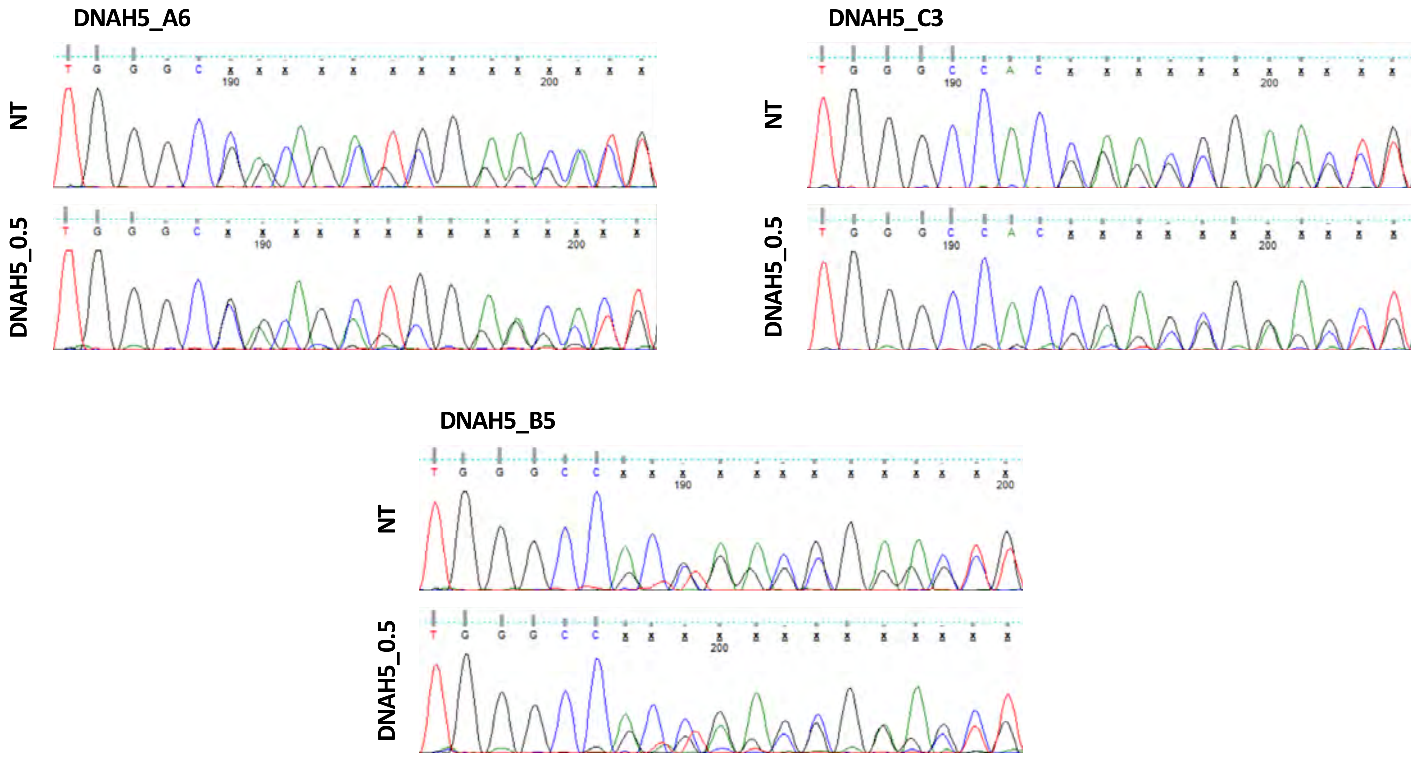

Supplementary Figure S11

a *Integrative lentiviral vector plasmids*

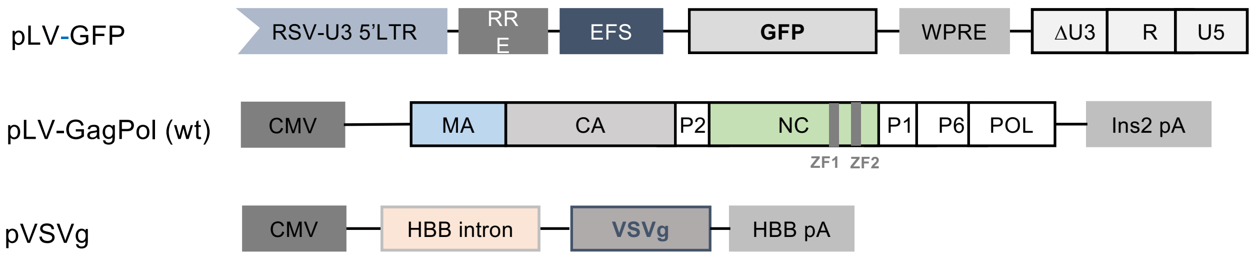

b *Non integrative LentiFlash® vector plasmids*

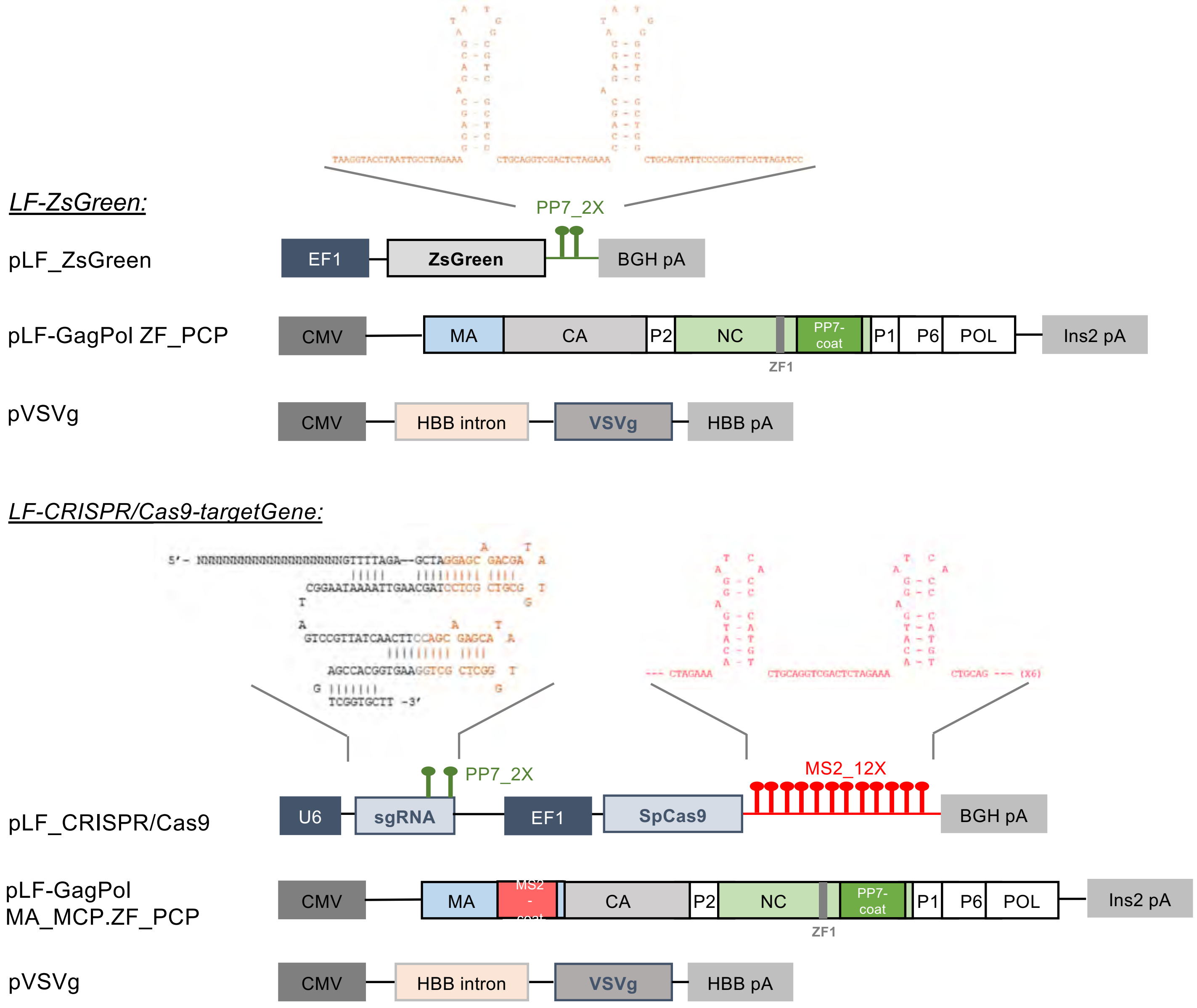

Supplementary Tables

Supplementary Table S1

sgRNA name	5'-3' sequence	PAM (5'-3')
GFP	GACCAGGATGGGCACCACCC	CGG
DNAH5	GGATGACGTTGGGCCACGAG	CGG
MCIDAS	GTAGCGAAGAGCAGTCAGCG	AGG
TRAC	GAGAATCAAAATCGGTGAAT	AGG
CXCR4	GAAGCGTGATGACAAAGAGG	AGG
CCDC40	CAGGTCTTGGTGTAGAGCGC	GGG

Supplementary Table S2

Names	5'-3' sequence	Used for
ddPCR_GFP_F	CTGCTGCCCGACAACCAC	ddPCR
ddPCR_GFP_R	TCACGAACTCCAGCAGGAC	ddPCR
ddPCR_GFP_probe	CCAGTCCGCCCTGAGCAAAGACC	ddPCR
ddPCR_CCDC40_YGT_F	CAGAAGGAGGAGGAGCTG	ddPCR
ddPCR_CCDC40_YGT_R	AAACCCTCCAGGACCG	ddPCR
CCDC40_YGT_probe	CGCTCTctACA	ddPCR
ddPCR_RPP30_F	TCAGCATGGCGGTGTTT	ddPCR
ddPCR_RPP30_R	GCTGTCTCCACAAGTC	ddPCR
ddPCR_RPP30_probe	TTCTGACCTGAAGGCTCTGCGC	ddPCR
GFP_F1	ATTGAACCGGTGCCTAGAGA	genotyping
GFP_R1	TTCATGTGGTCGGGGTAGC	genotyping
DNAH5_F4	AATGGATGCATGCTAAGTGAGTAA	genotyping
DNAH5_R4	GCCTCCAAAGTGATGTGAGGG	genotyping
DNAH5_F3	ACTTTAGCGTCTCCAGCCCC	genotyping
DNAH5_OT1_F2	TTTGATGCTCATCTGTCCTTTGA	genotyping
DNAH5_OT1_R2	GAAACTCAGCCGCTTTTTGTCT	genotyping
DNAH5_OT1_R1	CCAGTTCATTCATTCACTGGCT	genotyping
DNAH5_OT2_F	ACGAGTGTGCACAGGTACAG	genotyping
DNAH5_OT2_R	GCAGTCGGCCCTGTCTATTC	genotyping
DNAH5_OT3_F3	TGGGGATTTGGCCTAAGATG	genotyping
DNAH5_OT3_R3	AAACAGTCCTGCCTCGGGA	genotyping
MCIDAS_F1	TCTGACGTCCTAGCTGCG	genotyping
MCIDAS_R3	CACAATGCTTCCCCTCACCA	genotyping
MCIDAS_R2	ACAATGCTTCCCCTCACCAG	genotyping
MCIDAS_OT1_F	GGACGGTCAGCAGGCTACTT	genotyping
MCIDAS_OT1_R	TCAAGACCGGGATCACCACA	genotyping
MCIDAS_OT2_F	CACCTTGGCCATGTTGAAGC	genotyping
MCIDAS_OT2_R	CTGAGCTGGAGAAGCTGGAC	genotyping
MCIDAS_OT3_F2	TTGGTTCCCCGCGAAGTC	genotyping
MCIDAS_OT3_R2	TGAGAGACGGTATCCCCAGT	genotyping
MCIDAS_OT3_R1	AAGAGAAGAGTGGAGGGGCA	genotyping
MCIDAS_OT4_F	ACTGAGGAAAGGACTCACTGG	genotyping
MCIDAS_OT4_R	TTGTGGAAGTAGCGTAGCCA	genotyping
MCIDAS_OT5_F	GCAGAGGTGCTGAAACCTTC	genotyping
MCIDAS_OT5_R	AGTGGCTTGTTGATGCCCTT	genotyping
MCIDAS_OT6_F	AAAATGACCCATCCCCTGCAAA	genotyping
MCIDAS_OT6_R	TTTAAAACTGCTGCTGTAGGTGA	genotyping
TRAC_F2	TTGATAGCTTGTGCCTGTCCC	genotyping
TRAC_R2	GGCAAACAGTCTGAGCAAAGG	genotyping
TRAC_F1	TCACGAGCAGCTGGTTTCTAA	genotyping
CXCR4_F1	ATCTGCCTCACTGACGTTGG	genotyping
CXCR4_R2	TTCTCTTGTGCCCTTAGCCC	genotyping
CXCR4_R1	ATGGGCTCAGGGGACTATGA	genotyping
CCDC40_F2	GGCTGCCCTGAAGAACTACC	genotyping
CCDC40_R2	GCATTTGCTGCTTTGTCCTGA	genotyping
CCDC40_F3	AGCTGGGGGTGAATCTCTATGA	genotyping
CCDC40_H_F	GTGAATCTCTATGAGGTGCAGCAG	genotyping
DNAH5_HRMA_F2	GCATTTGCAGGTTCTTGCTG	HRMA
DNAH5_HRMA_R2	TCCAAAAGGTAGTTAAACTTGGAGA	HRMA
MCIDAS_HRMA_F2	GTCTCCCCGCGCAGC	HRMA
MCIDAS_HRMA_R2	CGGACCCGAGTAGCGAAG	HRMA
ZsGreen_F	TCTGCAACGCCGACATCA	qPCR
ZsGreen_R	GTTCACGCCGTAGAACTTGGA	qPCR
Cas9_F	AATGGCCTGTTCGGAAACCT	qPCR
Cas9_R	CTCAGCTGCAGTTTGGCATC	qPCR
GAPDH_F	GACCTGACCTGCCGTCTAGAAA	qPCR
GAPDH_R	CCTGCTTCACCACCTTCTTGA	qPCR
Cas9_F	AAACAGCAGATTCGCCTGGA	PCR (Fig S5e)
Cas9_R	TCATCCGCTCGATGAAGCTC	PCR (Fig S5e)
DNAH5_NGS_F	AATGATACGGCGACCACCGAGATCTACACTCGTGGAGCGTCGTCGG CAGCGTCAGATGTGTATAAGAGACAGGTGCAATGGCTCGTGTTTTT	NGS
DNAH5_NGS_R	CAAGCAGAAGACGGCATACGAGATCGCTCAGTTCGTCTCGTGGGCTC GGAGATGTGTATAAGAGACAGGGGCTTTTCAATTGTTCCAA	NGS

Supplementary Table S3

Immunofluorescence antibody name	Reference	Provider	dilution
OCT3/4	sc-9081	Santa Cruz Biotechnology	1/400
SSEA-4	90231	Chemicon	1/250
NANOG	ab109250	Abcam	1/200
SOX2	MA1-014	Invitrogen	1/100
FOXA2	af2400	RD system	1/100
SOX17	af1924	RD system	1/100
hNESTIN	MA1-110	Invitrogen	1/100
PAX6	mab5552	Chemicon	1/100
AlexaFluor 488 anti-rabbit IgG	A21206	Invitrogen	1/1000
AlexaFluor 555 anti-mouse IgG	A31570	Invitrogen	1/1000
AlexaFluor 647 anti-goat IgG	A21447	Invitrogen	1/1000
Flox cytometry antibody name	Reference	Provider	dilution
CXCR4-PE	557145	BD Biosciences	1/200
Isotype Ctr (PE) CXCR4	556653	BD Biosciences	1/200
